# The intratumoral microbiome in breast cancer: an actionable component of the tumor immune microenvironment

**DOI:** 10.3389/fimmu.2026.1944056

**Published:** 2026-10-06

**Authors:** Chiara Cossu, Manuel Adamo, Antonino Di Lorenzo, Laura Conti

**Affiliations:** Department of Molecular Biotechnology and Health Sciences, University of Turin, Turin, Italy

**Keywords:** breast cancer, chemotherapy, immunotherapy, microbiome, therapy resistance

## Abstract

Breast cancer remains one of the leading causes of cancer-related deaths in women. The discovery of a breast tumor microbiota has deeply changed our understanding of the breast tumor microenvironment, identifying microorganisms as active players in tumor progression. Increasing evidence suggests that intratumoral bacteria may influence multiple hallmarks of breast cancer, including chronic inflammation, immune modulation, metabolic reprogramming, epithelial plasticity, metastatic dissemination, and therapeutic response. Distinct microbial communities have been associated with specific breast cancer subtypes and clinical outcomes, and some mechanistic studies have demonstrated that individual bacterial species and their metabolites can directly regulate oncogenic signaling pathways, reshape the tumor immune microenvironment, and alter sensitivity to chemotherapy and immunotherapy. These findings have generated growing interest in exploiting the breast tumor microbiota as a source of novel diagnostic, prognostic, and predictive biomarkers, as well as a therapeutic target for breast cancer treatment. In this review, we summarize current knowledge on the composition and origin of the breast tumor microbiota, discuss the molecular mechanisms through which tumor-associated microorganisms may influence breast cancer initiation and progression, examine their impact on treatment response, and highlight emerging microbiota-based therapeutic strategies. Finally, we discuss the major technical and biological challenges that currently limit clinical translation, including low microbial biomass, contamination, lack of methodological standardization, and patient-related confounding factors, and outline future perspectives for integrating microbial profiling into precision oncology. A deeper understanding of host-microbe interactions within the breast tumor microenvironment may ultimately enable microbiome-informed approaches for personalized prevention, diagnosis, prognostication, and treatment of breast cancer.

## 1 Introduction

The human body is home to trillions of microorganisms, collectively referred to as the human microbiota, including bacteria, fungi (the mycobiota), and viruses. Their total number is estimated to be approximately equivalent to that of human cells in the body (1–3). While the term “microbiota” refers to the microbes themselves, the microbiome encompasses these microorganisms together with their collective genomes, and their “theater of activity”, including metabolites and surrounding environmental conditions (2, 4, 5).

Due to its extensive influence on host physiology and pathophysiology, the human microbiome, comprising both symbiotic and pathogenic microorganisms, is now considered as the body’s “last organ” (6). Microbial communities colonize multiple anatomical sites, such as the gastrointestinal tract, skin, oral cavity, and reproductive organs (7). Their composition is highly personalized, differing between individuals as uniquely as fingerprints. It also differs markedly across different body sites and over time (8). This microbial diversity and abundance are shaped by many factors, including diet, host genetics, disease status, pharmacological treatment, and lifestyle (9). Historically, many internal organs were considered sterile. However, recent technological advancements have revealed that tissues, including the lung, breast, liver, pancreas, prostate, and kidney, harbor low-biomass microbial communities (1, 2, 10). These resident microorganisms could play an important role in local homeostasis and disease progression. For example, the lungs, which constitute a barrier interface with the external environment, are enriched in bacterial genera such as *Modestobacter*, while *Propionibacterium* and members of the Enterobacteriaceae family are typically reduced (5). These commensal microbes can influence local immune responses, including the promotion of the expansion of γδ T cells, thereby promoting inflammation through the production of cytokines such as interleukin (IL)-17 and IL-23 (11, 12).

The colon harbors the highest microbial density in the human body, containing up to 10¹¹-10¹² microbial cells per gram of luminal content. It is predominantly populated by bacteria belonging to the phyla Bacteroidetes (Bacteroidota) and Firmicutes (recently renamed Bacillota) (5, 11, 13). Advances in high-throughput sequencing have revealed that the colon contains thousands of microbial species, making it one of the most densely populated microbial ecosystems known. Alterations in the relative abundance of these dominant phyla have been associated with several disorders, including inflammatory bowel disease, obesity, type 2 diabetes, and cancer (5, 11, 13). Importantly, the gut microbiome also exerts systemic effects on distant organs through the production of metabolites, modulation of immune responses, and translocation of microbial components. For instance, short-chain fatty acids (SCFAs) contribute to intestinal homeostasis by reducing inflammation and maintaining gut barrier integrity (14). On the other hand, other microbial-derived metabolites are associated with disease processes; for example, trimethylamine N-oxide (TMAO), generated through host metabolism of bacterially produced trimethylamine (TMA), has been linked to cardiovascular diseases (15), while elevated levels of certain secondary bile acids such as deoxycholic acid (DCA) and lithocholic acid (LCA) have been associated with colorectal cancer and other gastrointestinal disorders (16). Through the gut-liver axis, bacterial products are transported directly to the liver via the portal venous system, where they regulate metabolic and inflammatory pathways (17). Moreover, several bacterial molecules have been associated with disease development; for example, lipopolysaccharides (LPS) can trigger inflammatory responses, while the bacterial genotoxin colibactin has been linked to colorectal carcinogenesis (18). Consequently, alterations in gut microbial composition may influence the microbiota and physiological functions of extraintestinal tissues, including the liver, lungs, skin, and mammary gland.

Emerging evidence supports the existence of a gut-breast axis. Although the breast was long considered a sterile environment, molecular studies have demonstrated the presence of a distinct breast microbiota in healthy women. In one of the first comprehensive analyses, bacterial DNA was detected in breast tissue from 81 women undergoing breast surgery, with Proteobacteria representing the dominant phylum and genera such as *Acinetobacter*, *Pseudomonas*, *Staphylococcus*, and *Propionibacterium* among the most abundant taxa (19). Subsequent studies (summarized in **Table 1**) have shown that breast tissue microbial composition differs between healthy individuals and patients with breast cancer, suggesting a potential role for local microbial communities in carcinogenesis (5, 10, 20, 21). The mechanisms linking the gut and breast microbiomes are not yet fully understood and are thought to involve microbial translocation, immune modulation, and the activity of the estrobolome, the collection of gut microbial genes involved in estrogen metabolism. Through these pathways, gut microorganisms may influence systemic estrogen levels, inflammatory responses, and the breast tissue microenvironment, thereby contributing to breast health and disease (22, 23).

**Table 1:** Principal original studies characterizing the breast tumor microbiota (Reviews, general microbiome studies, studies conducted in other tumor types, and microbiota therapeutic intervention studies summarized in **Table 2** were not included).

Study	Species	Sample type	Breast cancer subtype	Microbial method	Main taxon/finding	Microbial/orthogonal validation	Functional/mechanistic validation	Contamination controls
Urbaniak et al., 2014 (19)	Human	Healthy breast tissue	N/A	16S rRNA V6 sequencing	Proteobacteria-dominant communities were detected in breast tissue; viable bacteria were cultured from all Canadian samples.	Aerobic/anaerobic culture of tissue homogenates; isolates identified by 16S Sanger sequencing.	None	Sterile PBS left open during surgery and processed in parallel (environmental control); nuclease-free-water PCR controls.
Xuan et al., 2014 (22)	Human	Paired tumor vs. normal adjacent tissue	ER-positive breast cancer	16S rRNA V4 sequencing	*Methylobacterium radiotolerans* was relatively enriched in tumors, whereas *Sphingomonas yanoikuyae* was more abundant in adjacent tissue; total bacterial load was lower in tumors.	Taxon-specific qPCR for *M. radiotolerans* and *S. yanoikuyae* and qPCR-based total bacterial load.	None	Not reported
Urbaniak et al., 2016 (21)	Human	Fresh breast tissue from healthy controls and women with benign or malignant disease (n=70)	Mixed	16S rRNA V6 sequencing	Cancer-associated tissue showed higher relative abundance of Bacillus, Enterobacteriaceae, and Staphylococcus than healthy tissue.	Culture and isolate identification by 16S Sanger sequencing (with biochemical confirmation of *E. coli*).	Breast isolates were co-cultured with HeLa cells; *E. coli* and *S. epidermidis* induced gamma-H2AX, supporting a genotoxic phenotype.	Open sterile PBS environmental control; disinfected-skin swab; nuclease-free water PCR controls; SourceTracker bioinformatic tool to assess contamination.
Hieken et al., 2016 (35)	Human	Aseptically collected breast tissue (invasive cancer vs. benign)	Predominantly ER/PR-positive invasive cancer	16S rRNA V3-V5 sequencing	Tissue adjacent to malignant disease differs from that of benign disease. Enrichment of low-abundance genera such as Fusobacterium, Atopobium, Hydrogenophaga, Gluconacetobacter, and Lactobacillus in tissue adjacent to malignant disease.	None	None	Aseptic operating sampling; skin tissue, skin and buccal swabs; empty tubes and clean swabs as negative controls.
Costantini et al., 2018 (46)	Human	Paired tumor vs normal breast tissue	Mixed	16S rRNA V2, V3, V4, V6 + 7, V8, and V9 sequencing	Ralstonia and Proteobacteria phylum were the most abundant. Tumor and paired normal tissues showed no significant richness difference; core needle biopsy and surgical excision produced comparable profiles.	None	None	Aseptic collection and separate sterile instruments; open TSB-buffer environmental controls processed with specimens.
Smith et al., 2019 (40)	Human	Breast tumors and matched adjacent-normal tissues, and 8 normal tissues from reduction mammoplasty	Mixed	16S rRNA V4 sequencing	Microbial communities differed by tissue type, race, stage, and subtype. Ralstonia was more abundant in tumors from non-Hispanic Black women, and TNBC was enriched in Streptococcaceae and Ruminococcus.	None	None	Aseptic collection and sterile-hood extraction; blank controls. Four template-free and four extraction-kit-reagent controls were co-sequenced; positive controls included.
Nejman et al., 2020 (33)	Human	Tumoral and adjacent tissues across seven tumor types, including breast	Mixed	5R 16S rDNA sequencing	Breast tumors showed comparatively high bacterial richness and diversity; bacteria were mainly intracellular in cancer and immune cells.	16S qPCR; 16S-rRNA FISH; LPS/LTA immunohistochemistry; electron microscopy; broad culture with isolate whole-genome sequencing.	None	437 extraction controls, 206 PCR no-template controls, and 168 paraffin-only controls; six-step contaminant filtering; assay-negative controls for imaging and culture.
Chiba et al., 2020 (90)	Human	Aseptically collected, snap-frozen tumors after neoadjuvant chemotherapy or without prior chemotherapy	Mixed	16S rRNA sequencing	Neoadjuvant chemotherapy increased tumoral *Pseudomonas* abundance and decreased *Streptococcus* populations. *Brevundimonas* and *Staphylococcus* were associated with distant metastasis.	*P. aeruginosa*-specific immunohistochemistry in an independent tissue microarray.	*P. aeruginosa* conditioned medium altered breast cancer cell growth, apoptosis, signaling and chemotherapy response *in vitro*.	Four template-free and four extraction kit reagent controls co-sequenced with specimens; two positive controls.
Parhi et al., 2020 (47)	Human and mouse	FFPE human breast tumors; murine breast cancer cell line	Mixed human breast cancer subtypes; TNBC mouse model	5R 16S rDNA sequencing	*F. nucleatum* colonized mammary tumors through Fap2-dependent binding and was linked to reduced T-cell accumulation, faster growth, and metastasis.	*F. nucleatum*-targeted qPCR/5R detection in human tumors; anaerobic culture from mouse tissues.	WT versus Fap2-deficient *F. nucleatum*, GalNAc competition, mouse tumor colonization, immune-cell analysis, tumor growth/metastasis and metronidazole treatment tested Fap2-dependent causality.	Fifty empty-extraction controls and 50 PCR no-template controls processed with 50 FFPE tumors.
Tzeng et al., 2021 (34)	Human	Fresh-frozen tumor, tumor-adjacent, high-risk, and healthy breast tissue	Mixed subtypes and stages	16S rRNA sequencing	Tumor tissues showed reduced bacterial diversity and distinct microbial profiles associated with clinicopathological features and local immune signatures.	16S qPCR to quantify absolute bacterial load.	None	Operating-room environmental controls; buffer-only extraction negatives; water PCR negatives; Zymo microbial-community positive controls.
Hadzega et al., 2021 (37)	Human	rRNA-depleted RNA-seq datasets from primary tumors and healthy breast samples	Mixed breast cancer subtypes and healthy tissue	Bioinformatic analysis of RNA-seq metatranscriptomics data	Proteobacteria, Firmicutes, and Actinobacteria are enriched; differences in transcript-derived microbial profiles were reported between tumor and healthy datasets and among selected clinical groups.	None	None	Synthetic human reads were used as a false-positive control and synthetic microbial reads as a positive classifier control.
Hogan et al., 2021 (119)	Human	Fresh breast tissue biopsies, breast tumors and matched normal breast tissues	Mixed	16S rRNA sequencing	Bacterial signatures distinguish tumor from matched normal tissue	None	None	Twelve extraction-kit negatives pooled into three sequencing libraries and two PCR no-template controls; SourceTracker and Decontam filtering; skin swabs and a progressive RIDE-based contamination-control workflow.
Banerjee et al., 2021 (36)	Human	FFPE breast tumors	Mixed	PathoChip pan-pathogen nucleic-acid hybridization after whole-transcriptome amplification	Specific microbial features are associated with different breast tumor subtypes; ER-positive tumors appeared most diverse and TNBC least diverse.	None	None	Sections were prepared in a sterile environment and the microtome sterilized between samples; reduction-mammoplasty/matched tissues were biological controls.
Parida et al., 2021 (72)	Human and mouse	Murine breast cancer cells and syngeneic mouse models	Mixed	Targeted detection of *B. fragilis*; ETBF colonization	ETBF/BFT activated β-catenin-Notch1 signaling and enhanced epithelial hyperplasia, tumor growth, and metastasis.	Targeted detection of *B. fragilis*/BFT in human and experimental material; in mice receiving intraductal bacteria, *B. fragilis* and BFT were confirmed by PCR. The supplement also shows histologic and immunostaining readouts in experimental tumors.	Purified BFT exposure in breast epithelial/cancer cells; inactive BFT-H352Y control; β-catenin and Notch1 inhibition with ICG001 and DAPT *in vitro* and *in vivo*; ETBF versus bft-deletion 086mut and sham controls; mammosphere, migration/invasion, tumor-growth, and metastasis assays.	Not reported
Fu et al., 2022 (13)	Human and mouse	Fresh murine primary tumors, circulating tumor cells, and lung metastases; fresh human tumors	Not specified	16S rRNA sequencing	Intracellular bacteria remodeled the actin cytoskeleton and increased survival of circulating tumor cells under mechanical stress.	V9 16S qPCR; aerobic/anaerobic culture with isolate identification; 16S FISH; LPS/LTA staining; microscopy and fluorescent/genetic bacterial tracing.	Antibiotic depletion, germ-free and conventional mouse experiments, bacterial re-administration/tracing, 3D organoid co-culture, fluid-shear-stress assays, and metastasis assays tested causality.	Sterile operating-room collection and clean-hood processing; PBS environmental background controls underwent the complete workflow; DEPC-water no-template controls and *E. coli* positive controls; mouse and human profiles underwent a control-based contamination correction.
Wang et al., 2022 (38)	Human and mouse	Fresh and frozen human TNBC samples, TNBC cell lines and syngeneic mouse models	TNBC	RNA sequencing	Clostridiales and related TMAO metabolite were enriched in immune-activated TNBC. TMAO activates pyroptosis in tumor cells, enhancing CD8^+^ antitumor immunity.	Universal 16S FISH confirmed bacterial signals in fresh TNBC tissues; targeted metabolomics quantified intratumoral TMAO. Clostridiales were inferred from PathSeq analysis rather than validated by organism-specific culture or imaging.	TMAO treatment, choline-rich diet, anti-PD-1 treatment, CD8 depletion, GSDME knockdown, and PERK inhibition tested the TMAO-PERK-pyroptosis-CD8+ immunity mechanism.	PathSeq human-read subtraction and microbial-reference mapping were used.
German et al., 2023 (10)	Human	Fresh-frozen normal breast cores from cancer-free women and tumor and/or tumor-adjacent tissue from breast cancer patients	Cancer-free breast tissue and mixed cancer subtypes	16S rRNA V1V2, V2V3, V3V4, V4V5, V5V7, and V7V9 sequencing	Lactobacillaceae, Acetobacteraceae, and Xanthomonadaceae were abundant in normal breast tissue, whereas Ralstonia was more abundant in tumors and tumor-adjacent tissue.	None	None	Environmental water/saline controls, extraction-kit and processor controls, microbial-community positive control; sterile/UV-clean workflow and Decontam filtering.
Bernardo et al., 2023 (80)	Mouse	Fresh murine breast cancer tissues	TNBC and HER2+	16S rRNA sequencing	Ampicillin reduced intratumoral *S. epidermidis*, favored an antitumor immune milieu, and slowed tumor growth.	Viable bacteria were cultured from tumors and isolates were identified to species level by MALDI-TOF; 16S copy number and V3-V4 profiles were compared between treatment groups.	Antibiotic treatment by different routes, transfer of tumor-derived *S. epidermidis*, *M. luteus*, or *C. perfringens*, macrophage co-culture, immune profiling, tumor-growth/metastasis assays, and ampicillin-paclitaxel treatment tested functional effects.	Tumors were collected under sterile conditions and laminar flow.
Ma et al., 2024 (93)	Human	Fresh and FFPE human TNBC samples and para-tumor tissue from patients treated with taxanes.	TNBC	16S rRNA sequencing	*Bacteroides fragilis* (ETBF) was enriched in poor responders; BFT-1 stabilized NOD1 and expanded ALDH-positive breast cancer stem-like cells through the NUMB-NOTCH1 axis.	BFT-gene qRT-PCR and ETBF-specific FISH in an independent pretreatment biopsy cohort.	ETBF/BFT-1 exposure, antibiotics/metronidazole, NOD1 inhibition/perturbation, and GAK–NUMB–NOTCH1 experiments in cells, xenografts, and PDX models tested chemoresistance.	Not reported
Guo et al., 2024 (97)	Human	*In vitro F. nucleatum*-tumor-immune cell co-culture	TNBC and ER+/PR+ breast cancer cell lines	F. nucleatum co-culture	*F. nucleatum* increased PD-L1 through NF-κB signaling and reduced CD8^+^ T-cell killing.	Not applicable	Untreated and *F. nucleatum*-treated groups, BAY11–7082 NF-kappaB inhibition, empty-vector controls, and PD-L1 knockdown were used in CD8+ T-cell killing and pathway assays.	Not applicable
Chen et al., 2025 (96)	Human	Pretreatment human tumor tissue from patients receiving neoadjuvant chemoimmunotherapy	Early TNBC	16S rDNA sequencing	Pathologic complete response was associated with higher bacterial load and diversity, more activated CD4^+^ T cells, and fewer immunosuppressive macrophages.	Absolute/relative 16S qPCR; 16S FISH; LPS IHC; aerobic/anaerobic culture with 16S sequencing of colonies; replication in an independent cohort.	None	Four control samples were included in the 16S workflow. Filtering addressed host-genome contamination and sequencing-lane, DNA-extraction, and PCR batches, followed by singleton-ASV and multi-patient-presence filters.
Liu et al., 2026 (81)	Human and mouse	Fresh-frozen tumors from breast cancer patients vs tissues from control patients without breast cancer. Tumors from TNBC mouse models	Mixed human breast cancer subtypes; murine TNBC models	16S rRNA sequencing, RNAscope-FISH	Intratumoral *Staphylococcus* correlated with cytotoxic CD8 T-cell activation; *S. aureus* depleted NAD metabolites and suppressed TNBC growth.	Bacterial 16S RNAscope-FISH in human tumors; species-level reanalysis and independent transcriptomic cohort.	Intratumoral *S. aureus* administration, CD8+ analyses/depletion, bacterial supernatant experiments, and metabolomics tested NAD depletion, immunity, and tumor growth.	Human 16S data were reused from Tzeng et al., including its environmental/extraction/PCR controls and contaminant-ASV subtraction; defined-strain mouse experiments were separate.
Klann et al., 2020 (31)	Human	Frozen breast tumors and bilateral normal breast tissue from reduction mammoplasty	Mixed	16S rRNA V1-V2 sequencing	Normal tissue showed higher diversity than tumor tissue; tumor profiles clustered separately, while normal-tissue microbiota varied by individual and breast side.	None	None	A sterile-water blank negative control was carried through DNA extraction, amplification, and sequencing; all OTUs detected in the blank were removed before analysis.
Hoskinson et al., 2022 (32)	Human	Healthy, prediagnostic, adjacent-normal, and tumor breast tissues	Mixed	16S rRNA V3-V4 sequencing	Prediagnostic tissue showed an intermediate compositional signature, including shifts in Bacillaceae, Burkholderiaceae, Corynebacteriaceae, Streptococcaceae, and Staphylococcaceae, that was more significant in adjacent-normal and tumor tissue.	None	None	Nine reagent extraction controls processed through library preparation/sequencing; positive microbial standard; prevalence/frequency-based Decontam filtering.
Meng et al., 2018 (42)	Human	Aseptically collected needle biopsies from benign and malignant breast lesions	Mixed	16S rRNA V1-V2 sequencing	Malignant tissue was enriched in Propionicimonas and Micrococcaceae, Caulobacteraceae, Rhodobacteraceae, Nocardioidaceae, and Methylobacteriaceae; Bacteroidaceae decreased and Agrococcus increased with histological grade.	None	None	Aseptic needle biopsy.
Laborda-Illanes et al., 2024 (43)	Human	Pretreatment FFPE breast tumors	Mixed	16S rRNA V2, V3, V4, V6-7, V8, and V9 sequencing	Tumors from patients who developed metastasis showed greater microbial richness/diversity and enrichment of *C. kroppenstedtii*, *C. aurimucosum*, and *H. parainfluenzae*. A five-species/two-miRNA signature predicted metastasis and was associated with overall survival.	None	None	The surface of FFPE blocks was removed; internal tissue was processed under sterile clean-bench conditions; the microtome and blades were rigorously cleaned between specimens. Sampling blanks, DNA-extraction blanks, and no-template amplification controls were included.
Todorovic et al., 2026 (44)	Human	FFPE tumors from metastatic and non-metastatic breast cancer with paired paraffin blanks	Not specified	16S rRNA V3-V4 sequencing	Distinct microbial signatures between metastatic and non-metastatic breast cancer; Clostridia and Burkholderiaceae were enriched in non-metastatic tumors. *Streptococcus* spp. predominated in metastatic tumors, *Neisseria* spp. in non-metastatic tumors.	None	None	First microtome slides discarded; microtome cleaned between specimens; paired blank paraffin from the same FFPE blocks collected and analyzed in parallel.
Zhao et al., 2025 (52)	Human	Fresh-frozen breast tumors	Not specified	5R 16S rRNA sequencing	*F. nucleatum* localized preferentially to tumor-cell-rich regions and was associated with MAPK and focal-adhesion alterations; co-culture promoted cancer-cell proliferation and migration, and VEGFD/PAK1 knockdown partially reduced these effects.	RNAscope-FISH/CISH with positive and negative probes; spatial transcriptomics/proteomics localized *F. nucleatum*-rich regions.	*F. nucleatum* co-culture, *E. coli* specificity control, proliferation/migration/invasion assays, and VEGFD/PAK1 siRNA knockdown tested downstream effects.	Blank, DNA-extraction, and no-template controls processed like tumor samples; RNAscope positive/negative probe controls; sterile grid dissection.

The balance of the breast microbial communities, known as symbiosis or eubiosis, can be disrupted, leading to a state known as dysbiosis (10, 20). This imbalance, characterized by harmful bacterial species outnumbering beneficial ones, might have negative effects on health and contribute to various pathological conditions, including neoplasia and tumorigenesis (20), influencing key cancer pathways (11). These microbes can originate from several potential sources, such as migration from the skin through nipple orifices, translocation from the gut via the proposed entero-mammary pathway involving immune cells and the lymphatic system, or hematogenous spread through the blood (2, 3, 24).

The study of tumor-associated microbiota or tumor microbiota, defined as the collection of microorganisms found within tumor tissues (1, 3), is an emerging field. Intratumoral microorganisms have been identified in at least 33 major cancer types and constitute a key component of the tumor microenvironment (TME), often found within immune cells (1). Regarding breast cancer, the local breast microbiota is now considered an integral component of the tumor immune microenvironment (TIME). Microorganisms can influence the density and functional state of both antitumor and immunosuppressive cell populations, thereby modulating the local “immune tone” (3, 12). For instance, bacteria such as *Lactococcus* and *Streptococcus* are abundant and may have a protective role by activating natural killer (NK) cells or producing antioxidant metabolites that prevent DNA damage (3, 20, 25). However, the precise causal roles and complex interactions between fungi, bacteria, and the host in tumorigenesis require further investigation.

The aim of this review is to integrate and synthesize current knowledge on the interplay between the microbiome, with particular emphasis on the breast microbiome tumor cells, and the immune system. We focus on how these interactions may influence cancer onset and progression, as well as the development of chemotherapy and immunotherapy resistance. Understanding these mechanisms could provide new opportunities for patient stratification and the development of microbiome-based interventions for precision immuno-oncology.

## 2 Literature search strategy

Literature was identified through searches in PubMed/MEDLINE, Embase, and Scopus, covering articles published in the last 15 years. No date restriction was applied to background literature on general microbiome biology, host-microbe interactions and microbiome methodology, or to seminal mechanistic studies in other tumor types. Search terms were combined and included “breast cancer”, “microbiota”, “intratumoral microbiome”, “microbiome”, “microbial communities”, “tumor microenvironment”, “immunotherapy”, “targeted therapy”, “therapy resistance”, “chemotherapy”, “low-biomass” and “guidelines”. Eligible studies were peer-reviewed, English-language articles available in full text. Clinical studies in adult women with breast cancer were prioritized. Preclinical *in vitro* studies and animal models were also included, as were seminal mechanistic studies in other tumor types. Titles and abstracts were screened independently by the authors, and full texts were assessed for relevance. Any disagreements arising during the selection process were resolved through discussion among all the authors.

## 3 The breast microbiota

Breast cancer is the second most frequently diagnosed malignancy among women worldwide and remains a leading cause of cancer-related mortality. According to GLOBOCAN estimates, approximately 2.4 million new cases and 670,000 deaths were recorded globally in 2024 (26). Despite significant advances in early detection, molecular classification, and targeted therapies, breast cancer remains a highly heterogeneous disease characterized by considerable variability in clinical behavior, therapeutic response, and patient outcomes (27). This heterogeneity is reflected in the classification of breast cancer into distinct molecular subtypes, including luminal A, luminal B, HER2-positive, and triple-negative breast cancer (TNBC), each displaying unique biological and immunological features (28). Traditionally, breast cancer progression has been attributed primarily to tumor-intrinsic genetic and epigenetic alterations. However, increasing evidence indicates that the TME plays a critical role in tumor initiation, progression, metastatic dissemination, and therapeutic resistance. The breast TME consists of a dynamic and complex network of cancer cells, immune cells, cancer-associated fibroblasts, adipocytes, endothelial cells, and extracellular matrix components that continuously interact through cytokines, chemokines, growth factors, and metabolic signals. These interactions shape tumor evolution and profoundly influence antitumor immune responses, and the immune landscape of breast cancer has emerged as a major determinant of prognosis and treatment efficacy (29).

High levels of tumor-infiltrating lymphocytes (TILs), particularly CD8^+^ cytotoxic T cells, are associated with improved clinical outcomes and enhanced responses to chemotherapy and immune checkpoint blockade, especially in TNBC and HER2-positive tumors. Conversely, the accumulation of immunosuppressive cell populations, including regulatory T cells (Tregs), myeloid-derived suppressor cells (MDSCs), and tumor-associated macrophages (TAMs), promotes immune evasion and disease progression. As a result, increasing attention has focused on understanding the factors that regulate the immune composition and functional state of the breast TME (28). In recent years, both local microbial communities residing within breast tissue and the gut microbiome have been shown to modulate systemic and local immune responses through the production of metabolites, regulation of inflammatory pathways, and shaping of immune cell differentiation and activation. These observations have led to the hypothesis that microbiome-host interactions may contribute to the establishment of distinct immune microenvironments within breast tumors and influence responsiveness to immunotherapeutic strategies.

It is well documented that distinct microbial compositions characterize malignant tissues compared with healthy counterparts within different organs (2, 10). The healthy breast harbors a distinct microbiota and, although a universally accepted core breast microbiota has not yet been established, healthy breast tissue is generally enriched in members of the phyla Proteobacteria, Firmicutes, Actinobacteria, and Bacteroidetes (10). Several studies (summarized in **Table 1**) have reported the presence of bacterial families including Lactobacillaceae, Acetobacteraceae, Xanthomonadaceae, Enterobacteriaceae, Staphylococcaceae, and Comamonadaceae, although their relative abundance varies substantially across cohorts and sequencing methodologies (10, 19, 30). For example, early work by Urbaniak et al., performed on fresh or snap-frozen samples by both 16S rRNA DNA sequencing and *in vitro* cultures, reported Proteobacteria as the dominant phylum in breast tissue, with abundant *Bacillus, Acinetobacter*, Enterobacteriaceae, *Pseudomonas, Staphylococcus, Propionibacterium*, Comamonadaceae and *Prevotella* (19). Klann et al. sequenced 36 frozen bilateral normal breast-tissue samples from ten women undergoing mammoplasty and found that Bacteroidetes, Firmicutes, Proteobacteria and Actinobacteria were the dominant phyla; they also discovered substantial variation between women and between the two breasts of the same woman (31). More recently, German et al. analyzed fresh-frozen normal breast tissue from 403 cancer-free women, together with tumor and histologically normal tumor-adjacent tissue from 76 patients with breast cancer. They identified Lactobacillaceae, Acetobacteraceae and Xanthomonadaceae as dominant families, with *Acetobacter aceti, Lactobacillus vini, Lactobacillus paracasei* and *Xanthomonas* sp. enriched in normal breast tissue, whereas *Ralstonia* was more abundant in both breast tumors and histologically normal adjacent tissue.

Comparisons of breast tumors with adjacent normal tissue have yielded non-uniform signatures (**Table 1**): using non-sterile formalin-fixed tissue, Xuan et al. found *Methylobacterium radiotolerans* relatively enriched in tumor tissue and S*phingomonas yanoikuyae* enriched in paired normal adjacent tissue (22), whereas Urbaniak et al. reported that normal adjacent tissue and tumor tissue from the same patient were microbiologically similar, but differed from tissue from healthy controls, with higher Enterobacteriaceae, *Bacillus*, and *Staphylococcus* in women with breast cancer (21). Of note, in these studies interindividual differences were greater than differences among tissues from the same patient, raising the possibility that breast dysbiosis may precede tumor formation and contribute to a cancer-permissive microenvironment rather than developing exclusively because of tumor growth. Hoskinson et al. provided further support for this hypothesis by identifying an intermediate microbial profile in histologically normal breast tissue donated by women before their subsequent breast cancer diagnosis. Although these pre-diagnostic samples remained more similar to healthy tissue than to established tumors, they exhibited alterations in Bacillaceae (Firmicutes) and Burkholderiaceae (Proteobacteria) resembling those detected in normal-adjacent and tumor tissues. Conversely, Xanthobacteraceae was more abundant in breast tissue from healthy donors (32). These findings indicate that histologically normal tumor-adjacent tissue can harbor dysbiotic alterations potentially involved in carcinogenesis and that cancer-associated microbial changes may already be detectable before clinical diagnosis. Therefore, the most consistent distinction may not be between the tumor and its immediately adjacent tissue, but rather between the entire breast microenvironment of women with cancer and that of women without malignancy, suggesting that normal adjacent tissue should not be treated as equivalent to breast tissue from healthy donors.

Several studies have shown that breast tumors harbor bacterial loads up to tenfold higher than those of normal breast tissue (2), although Xuan et al. reported an approximately tenfold lower bacterial burden in tumors and a further inverse association between intratumoral bacterial load and disease stage (22). Moreover, breast cancer tissue generally shows reduced microbial α-diversity compared with genuinely normal breast tissue, even if it possesses the highest microbiota heterogeneity among tumor types (33, 34). Recently, Rad et al. conducted a systematic review and meta-analysis of 11 studies comprising 1,260 fresh or fresh-frozen breast tissue samples, including normal, benign, mastitis-affected, cancer-adjacent, and malignant tissues. Their standardized reanalysis of 16S rRNA sequencing datasets identified Proteobacteria, Firmicutes, Actinobacteriota, and Bacteroidota as the predominant phyla across breast tissue types, whereas *Staphylococcus* and *Corynebacterium* were among the most consistently detected genera. Cancer and cancer-adjacent tissues displayed broadly similar microbial profiles and lower α-diversity than normal and mastitis-affected tissues, although substantial variability was attributable to differences in primer sets and study methodologies. Analysis of TCGA-BRCA data further associated higher intratumoral abundances of *Staphylococcus* and *Corynebacterium* with proliferation-related transcriptional signatures; notably, high *Staphylococcus* abundance was associated with poorer overall survival (30). However, different studies gave different results. Indeed, by analyzing fresh-frozen breast tissue from 308 women, including 221 patients with breast cancer, 18 cancer-free women at increased genetic or clinical risk, and 69 healthy controls by 16S rRNA gene sequencing, Tzeng et al. showed that Proteobacteria predominated across all groups, but tumors showed increased relative abundances of Pseudomonadaceae and Enterobacteriaceae and of the genera *Pseudomonas*, *Proteus*, *Porphyromonas*, and *Azomonas*. Conversely, *Propionibacterium* and *Staphylococcus* were prominent in healthy, high-risk, and tumor-adjacent tissues but depleted in tumors, while *Stenotrophomonas* and *Caulobacter* were particularly characteristic of healthy controls. Specific microbial taxa were also associated with tumor stage, grade, histological subtype, receptor status, lymphovascular invasion, and nodal involvement. Integration with immune gene-expression and cytokine data further revealed distinct microbiome-immune association networks across tissue types, suggesting that local bacterial communities may be linked to the immunological state of breast tumors, although the observational design did not establish causality (34). Several primary studies have reported breast cancer-associated enrichment of potentially pro-inflammatory or pathogenic taxa, including *Fusobacterium*, *Lactobacillus* and *Methylobacterium radiotolerans* (22, 35). Higher relative abundances of *Bacillus*, *Staphylococcus*, and Enterobacteriaceae were detected in breast tissue from patients with cancer compared with healthy controls (21). However, these associations remain correlative and require validation through standardized profiling and functional studies.

Microbial composition has been reported to differ, in an associative manner, by tumor subtype in human cohort studies (**Table 1**). In a comprehensive profiling study on FFPE samples, Banerjee et al. reported that hormone receptor-positive (HR+) tumors displayed the highest microbial diversity, whereas HER2-positive and TNBC showed progressively lower diversity, with TNBC exhibiting the least complex microbiota and the lowest abundance (36). HER2-positive tumors appear to possess a distinct microbial signature characterized by the enrichment of several subtype-specific taxa. Genera including *Granulicatella*, *Filibacter*, *Anaerostipes*, *Dyadobacter*, *Cloacibacterium*, and *Alloprevotella* have been reported at higher abundance in HER2-positive tumors compared with other subtypes (33, 34). In addition, an overrepresentation of Proteobacteria-related taxa, including *Serratia*, members of the order Burkholderiales, and *Helicobacter pylori*, has been described in HER2-positive compared with HER2-negative disease (37). In TNBC, Proteobacteria is among the dominant phyla. At finer taxonomic resolution, TNBC often shows enrichment of Clostridiales (38) and has been associated with higher levels of *Arcanobacterium* and *Corynebacterium* (Actinobacteria), as well as *Achromobacter* and *Pseudomonas* (Proteobacteria) (21). A recent meta-analysis reprocessing 16S rRNA sequencing data from four independent cohorts showed that TNBC samples displayed reduced microbial richness and a distinct community composition, including lower abundances of Bacteroidetes and Firmicutes. Several taxa were enriched in TNBC, particularly Azospirillaceae and the genera *Azospirillum* and *Anaerobutyricum*. At the species level, *Gemmiger formicilis*, *Anaerobutyricum soehngenii*, and *Acinetobacter indicus* were enriched, whereas *Anaerococcus nagyae*, *Corynebacterium pseudodiphtheriticum*, and *Novosphingobium nitrogenifigens* were depleted (39). In contrast, Luminal A and Luminal B HR+ tumors are enriched in members of the Firmicutes and Tenericutes phyla and frequently contain genera such as *Lactobacillus*, *Streptococcus*, and *Clostridium* (40). Firmicutes and Bacteroidetes have been proposed to interact with estrogen-dependent metabolic pathways, potentially contributing to a TME that supports tumor cell survival and growth (41). Among these taxa, *Lactobacillus* has attracted particular interest because of its ability to metabolize estrogens. This metabolic activity may influence local hormone availability within the TME and could affect the progression of hormone-driven cancers as well as their response to endocrine therapies. However, whether breast-resident strains directly metabolize estrogens and alter local hormone availability has not been established (21).

Beyond these subtype-associated differences, emerging evidence suggests that intratumoral microbial composition may also correlate with tumor stage and metastatic progression. Available comparisons suggest that benign breast disease may represent a microbiologically distinct condition rather than simply an intermediate state between healthy tissue and breast cancer (**Table 1**); however, a consistent taxonomic profile has not yet been established. In the study by Hieken et al., breast tissue from women with benign non-atypical disease and tissue adjacent to invasive breast cancer were both dominated by Bacteroidetes and Firmicutes and showed no significant difference in α-diversity. Nevertheless, their community structures differed significantly according to unweighted UniFrac analysis, indicating that the principal differences involved rare or low-abundance taxa rather than the dominant phyla. In particular, *Fusobacterium, Atopobium, Gluconacetobacter, Hydrogenophaga*, and *Lactobacillus* were enriched in breast tissue from patients with invasive cancer compared with women with benign disease (35). Importantly, however, these associations were based on nominal significance without correction for multiple comparisons and were obtained from tissue adjacent to the lesions rather than from direct sequencing of the benign and malignant lesions themselves. Meng et al. examined 94 aseptically collected percutaneous breast-tissue biopsies from a Chinese cohort, including 22 benign lesions and 72 malignant tumors. 16S rRNA gene sequencing showed a higher relative abundance of Proteobacteria in malignant tumors and identified *Propionicimonas* and the families Micrococcaceae, Caulobacteraceae, Rhodobacteraceae, Nocardioidaceae, and Methylobacteriaceae as taxa discriminating malignant from benign disease. Within the malignant subgroup, Bacteroidaceae decreased and the genus *Agrococcus* increased with increasing histological grade. Inferred functional profiling using PICRUSt also suggested differences in biotin and glycerophospholipid metabolic pathways; grade III tumors additionally displayed increased pathways related to ribosome biogenesis and decreased flavonoid biosynthesis (42). Thus, current evidence supports a taxonomic shift between benign and malignant breast disease but does not yet define a reproducible “benign breast microbiome.” Similarly, evidence for taxonomic differences specifically associated with metastatic breast cancer is considerably limited and, since breast cancer subtype is itself strongly associated with metastatic behavior, its potential confounding effect should be carefully considered when interpreting these data. Most human studies have analyzed primary tumors and have not included paired distant metastases; consequently, a reproducible taxonomic signature distinguishing primary non-metastatic tumors from metastatic lesions has not yet been established. Overall, the available studies indicate that the microbiota associated with breast tissue varies according to the subsequent development of metastasis. However, direct comparisons among microbial composition should be made cautiously, because the analytical platforms, study populations, and the nature of the specimens differ substantially across studies. Laborda-Illanes et al. used FFPE primary-tumor tissue collected during surgery, before systemic treatment and before clinical evidence of metastasis, from a cohort of 116 breast-cancer patients: 42 non-metastatic patients and 74 patients who subsequently developed metastases. Their model was subsequently assessed in an independent cohort of 35 patients. Tumor microbiota profiling by 16S rRNA sequencing, integrated with quantitative PCR analysis of tumor miRNAs, revealed greater microbial richness and diversity, a distinct community composition, and increased Fusobacteria in the metastatic group. At species level, *Alistipes onderdonkii*, *Haemophilus parainfluenzae*, *Micrococcus luteus*, *Corynebacterium aurimucosum*, *Corynebacterium kroppenstedtii*, *Staphylococcus epidermidis*, *Prevotella copri*, *Rothia dentocariosa*, *Rothia mucilaginosa*, and *Neisseria subflava* were more abundant in metastatic cases. Conversely, *Blautia obeum*, *Parabacteroides distasonis*, *Lactobacillus iners*, *Faecalibacterium prausnitzii*, *Bifidobacterium longum*, and *Bifidobacterium adolescentis* were enriched in non-metastatic cases. A combined signature comprising five bacterial species and two miRNAs significantly discriminated the two groups, although these observations remain preliminary associations rather than evidence of causality (43). Another recent study provided preliminary human evidence by profiling the microbiota of FFPE primary breast cancer specimens stratified according to metastatic status. No significant differences in α- or β-diversity were detected between metastatic and non-metastatic cases. However, differential-abundance analysis showed that the class Clostridia and the family Burkholderiaceae were significantly enriched in non-metastatic tumors, whereas microbial-network analysis identified *Anaerobacillus* as the principal hub in metastatic breast cancers and *Granulicatella* in non-metastatic tumors (44). These findings suggest that metastatic progression may be associated with changes in specific taxa and microbial community organization rather than with broad alterations in overall diversity. Nevertheless, they should be interpreted cautiously because the analysis included only 20 breast cancer specimens, comprising 8 metastatic and 12 non-metastatic cases (44). Moreover, the use of archival FFPE samples may introduce DNA degradation and contamination-related biases, particularly when studying low-biomass microbial communities. Current mechanistic evidence is therefore still largely preclinical and suggests that metastasis may be associated with the selective retention or enrichment of specific tumor-resident bacteria rather than with a uniform, high-level phylum shift. In the MMTV-PyMT mouse model, primary tumors contained predominantly Gram-positive facultative anaerobes belonging to the Firmicutes phylum, including *Enterococcus, Staphylococcus, Streptococcus*, and *Lactobacillus*. The same bacterial strains were detected in circulating tumor cells and pulmonary metastases, suggesting that intracellular bacteria can disseminate together with malignant cells, as discussed in the next Sections (13). More recently, comparison of highly metastatic 4T1 and non-metastatic 67NR mammary tumors identified overlapping bacterial genera but distinct species-level profiles, with several *Bacillus* species associated with metastatic tumors. In particular, *Bacillus thermoamylovorans*, isolated from metastatic tumors, increased metastatic burden, whereas *Bacillus subterraneus*, isolated from non-metastatic tumors, did not (45). These observations provide mechanistic evidence that individual bacterial species or strains may facilitate metastatic dissemination; however, they should not yet be interpreted as defining a human metastatic breast cancer microbiome.

Overall, breast carcinogenesis appears to be associated with changes in community composition, microbial load, and diversity, but the reported “cancer-associated” taxa differ substantially across cohorts. Healthy breast tissue, benign disease, primary cancer, and metastatic disease therefore cannot currently be assigned universally reproducible taxonomic profiles. Differences in tissue source, tumor subtype and stage, geographical population, 16S rRNA variable region, sequencing platform, bioinformatic pipeline, contamination-control procedures, and definitions of “normal” tissue all contribute to this variability.

The mechanisms through which microorganisms reach and colonize breast tissue remain not fully understood, although it is evident that tumor-associated microorganisms can access and colonize the breast through multiple routes. Current evidence indicates that bacteria may reach breast tissue through systemic dissemination via the bloodstream or lymphatic circulation, potentially translocating from the gut, oral cavity, or skin microbiota (33, 46). The presence of bacterial DNA and viable microorganisms in both healthy and malignant breast tissue supports the existence of these trafficking mechanisms, although their relative contribution remains unclear. Tumor vasculature, characterized by aberrant architecture and high permeability, further facilitates this process through specific adhesion mechanisms (17). For example, *Fusobacterium nucleatum* can disseminate through the bloodstream and bind to D-galactose-β (1–3)-N-acetyl-D-galactosamine (Gal-GalNAc) residues overexpressed in breast cancer through its Fusobacterium autotransporter 2 (Fap2) protein (47, 48). Moreover, different *Escherichia coli* strains can disrupt the gut vascular barrier, enabling systemic spread (49). Although the origin of breast-resident *Escherichia coli* has not been directly established, hematogenous translocation from the gut represents a plausible route. Indeed, mammary tumors can impair intestinal barrier integrity and promote systemic dissemination of enteric bacteria (50). Importantly, tumors themselves may attract microorganisms due to their immunosuppressive, hypoxic, and nutrient-rich environment (17, 51).

Emerging spatial studies indicate that intratumoral microbial communities in breast cancer are not uniformly distributed, although direct, region-matched comparisons of taxonomic diversity and absolute microbial load between the viable outer tumor rim and the hypoxic inner core remain limited. Zhao et al. performed 5R 16S rRNA gene sequencing on 60 spatially distinct tumor-tissue samples obtained from 15 patients with invasive breast cancer, identifying 5,757 operational taxonomic units and marked intratumoral compositional heterogeneity in 13 of 15 patients. Bacteroidota, Firmicutes, Proteobacteria, and Fusobacteriota were the predominant phyla, whereas *Prevotella* and *Fusobacterium* were the most abundant genera. Targeted RNAscope-FISH/chromogenic *in situ* hybridization in selected patient samples confirmed that *Fusobacterium nucleatum* displayed a focal and clustered distribution, predominantly in tumor-cell-rich regions, with sharp boundaries between *F. nucleatum*-positive and -negative areas. Although this study did not formally compare tumor core and invasive edge, *F. nucleatum* was observed in necrotic cavities and vascular regions in a subset of tumors, suggesting possible adaptation to hypoxic/necrotic niches and/or hematogenous dissemination (52). Complementary evidence was provided by Wang et al., who developed the miCDaL whole-tumor imaging method, combining fluorescent D-amino-acid labeling of metabolically active bacteria, modified iDISCO-CUBIC tissue clearing, and light-sheet fluorescence microscopy. In spontaneous MMTV-PyMT murine mammary tumors, this approach enabled centimeter-scale three-dimensional visualization and volumetric quantification of tumoral microbiota relative to tumor vasculature and necrotic regions. At early stages, only a small fraction of bacterial signals co-localized with scattered necrotic areas. In contrast, in more advanced tumors with larger centrally located necrotic cores, bacterial-colonized regions were enclosed by the necrotic compartment, indicating preferential localization in, or immediately adjacent to, necrotic regions that are generally hypoxic. The volume of bacteria-colonized tissue increased in parallel with tumor progression and expansion of necrosis, supporting an association between increased microbial burden and the development of hypoxic/necrotic tumor architecture. Notably, bacteria also accumulated in discrete areas near the tumor border, and bacteria-colonized regions were spatially segregated from activated T-cell compartments; in human breast-cancer samples, bacterial regions similarly showed minimal overlap with tertiary lymphoid structures and NK cells (53). Spatial analyses in colorectal and oral cancers have similarly shown that bacteria-rich microniches are less vascularized, less proliferative, and more immunosuppressive than adjacent bacteria-negative regions, supporting the preferential persistence of bacteria in poorly oxygenated breast tumor compartments (54). Thus, these data support a model in which breast tumor microbiota occupy spatially restricted, immunologically distinct niches, including regions associated with necrosis and likely hypoxia, but further human studies are needed to establish whether specific taxa and absolute bacterial loads consistently differ between the hypoxic core, viable tumor periphery, and invasive front.

### 3.1 Role of the breast microbiome in tumorigenesis

The microbiome might contribute to cancer initiation, progression, and metastasis through several mechanisms (1, 12) (**Figure 1**). Integrative genomic analyses have shown that specific bacterial taxa correlate with pathways involved in epithelial-to-mesenchymal transition (EMT), cell proliferation, and cancer-related metabolic reprogramming, suggesting that the breast tumor microbiome may contribute to shaping tumor cell phenotypes beyond its effects on inflammation and immunity. In particular, Thompson et al. found that in breast cancer specimens, *Listeria fleischmannii* was associated with EMT-related gene expression, while *Haemophilus influenzae* correlated with proliferative pathways, including the G2M checkpoint, E2F targets, and mitotic spindle assembly. Extending these observations through integrated bulk and single-cell analysis, Chen et al. identified microbial profiles associated with distinct tumor metabolic states, immune-cell composition, T-cell exclusion, treatment response, and overall survival. High *Campylobacter* abundance combined with elevated inositol phosphate metabolism was associated with poorer survival. However, both studies were based predominantly on computational analysis of sequencing-derived microbial reads and therefore establish correlations rather than direct causal relationships (55, 56).

**Figure 1:**
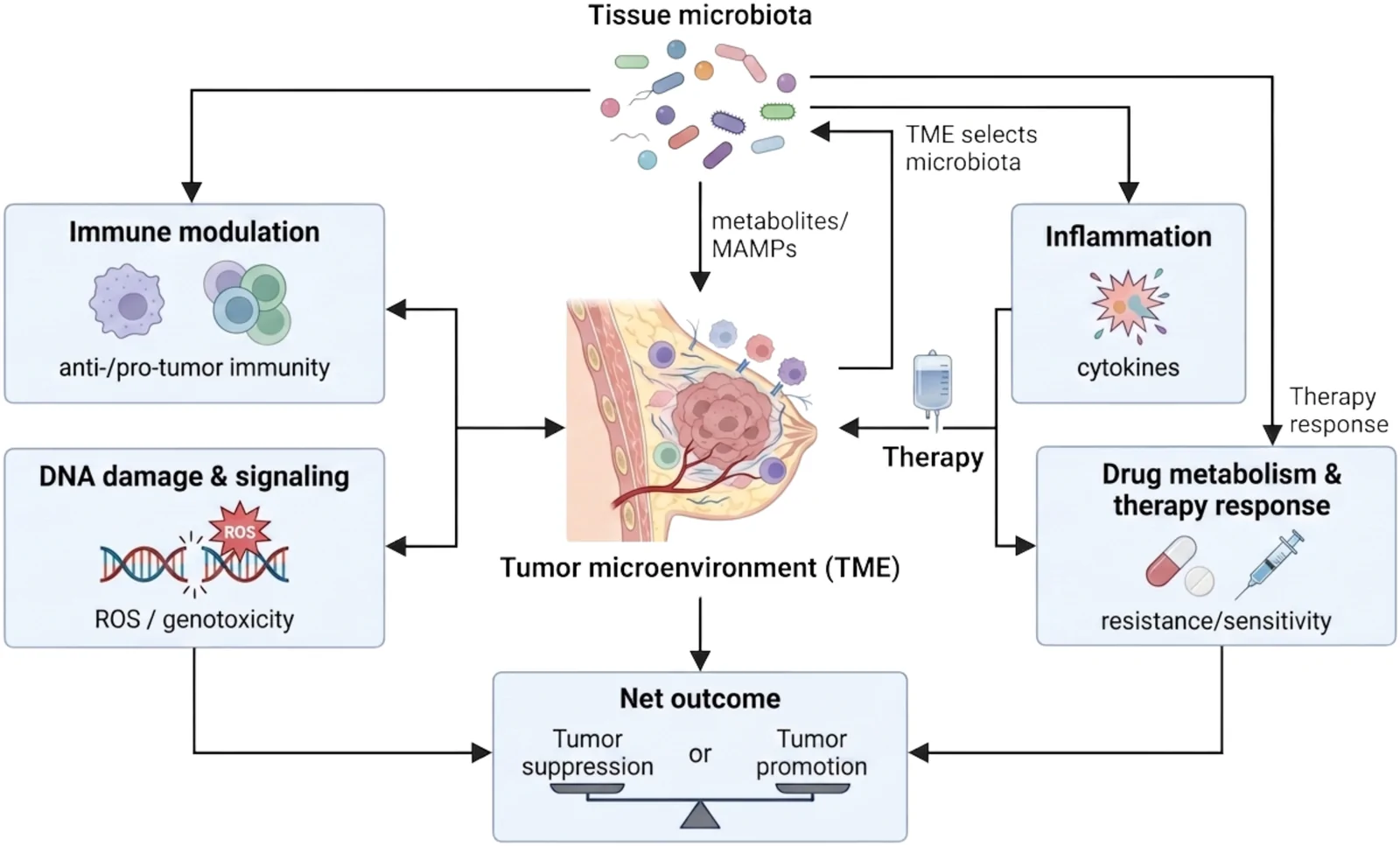
The breast tissue microbiome tips the balance between tumor suppression and tumor promotion through multiple mechanisms. Schematic representation of the bidirectional interaction between the tissue microbiome and the breast tumor microenvironment (TME). Bacteria residing within breast tissue release metabolites and microbe-associated molecular patterns (MAMPs) that act on the TME through four main routes: i) immune modulation, resulting in either anti- or pro-tumor immunity; ii) inflammation, through the induction of the production of cytokines; iii) DNA damage and signaling, through reactive oxygen species (ROS) and genotoxicity; and iv) drug metabolism, influencing therapy resistance or sensitivity. In turn, the TME selects the microbial community, while antitumor therapies act both on the tumor and on the microbiota, further reshaping its composition. The convergence of these mechanisms determines the net outcome, which may shift the balance either toward tumor suppression or tumor promotion. Created with BioRender.com.

At early stages of tumorigenesis, intratumoral bacteria may induce genomic instability through genotoxins, such as colibactin produced by *pks*+ *Escherichia coli*, reactive oxygen species, or other bacterial toxins (5, 17, 51, 57). Moreover, studies in gastric and intestinal models showed that *Helicobacter pylori* and enteropathogenic *Escherichia coli* interfere with host DNA repair pathways (58, 59). Tumor-associated bacteria might also modulate host epigenetic reprogramming, as suggested by the correlation between intratumoral *Fusobacterium nucleatum* and CDKN2A methylation observed in colorectal cancer (60). However, although breast-derived *Escherichia coli* and *Staphylococcus epidermidis* isolates can induce DNA double-strand breaks in mammalian cells *in vitro* (21), direct involvement of these mechanisms in breast carcinogenesis remains to be established.

Many microbial ligands bind pattern recognition receptors (PRRs), including Toll-like receptors (TLRs), leading to NF-kB activation and the establishment of a tumor-permissive environment (51). These effects do not require bacterial internalization, as extracellular or surface-associated microorganisms can act through direct adhesion to cancer cells or through the release of microbe-associated molecular patterns (MAMPs), toxins, metabolites, enzymes, and extracellular vesicles. For example, lipopolysaccharide derived from Gram-negative members of the breast microbiota promoted breast cancer growth through TLR4-dependent induction of S100A7 (61). Of note, we have demonstrated that breast cancer cells express TLR2 that, by binding danger-associated molecular patterns (DAMPs) released actively by cancer stem cells or passively by dying tumor cells, promotes cancer cell proliferation and survival (62, 63). A similar effect may be driven by TLR2 activation induced by MAMPs, thus mediating the pro-tumorigenic effects of some bacteria that may be enriched in the breast microbiota (64, 65). In parallel, bacteria can facilitate immune evasion by recruiting immunosuppressive cells such as MDSCs, Tregs, and M2-like macrophages (17), as discussed in the next section.

Microorganisms may facilitate tumor invasion and metastatic dissemination by altering cell-cell and cell-matrix adhesion and activating host proteolytic and stromal programs involved in extracellular matrix (ECM) remodeling. Although direct evidence for this mechanism in breast cancer remains limited, mammary models provide several indications that tumor-associated bacteria can induce molecular processes linked to ECM reorganization. In particular, *Fusobacterium nucleatum* induced inflammatory and hyperproliferative lesions and promoted tumor growth and metastatic progression, accompanied by enrichment of Wnt/β-catenin, MYC, KRAS, mTORC, and EMT-related programs (66). Spatial transcriptomic and proteomic profiling further showed that *F. nucleatum*-colonized epithelial tumor regions were enriched for proteins and pathways associated with proliferation, migration, invasion, focal adhesion, necroptosis, and MAPK signaling; VEGFD and PAK1 were identified as candidate mediators of these localized pro-tumor effects (52). More direct mechanistic evidence comes from colorectal cancer, where *Fusobacterium nucleatum* can promote EMT and the expression of chemokines, matrix metalloproteinases (MMPs) like MMP9 and MMP2, and adhesion molecules such as intercellular adhesion molecules (ICAM)-1, facilitating tumor cell adhesion to endothelial cells and extravasation (67, 68). Consistent with a potentially similar mechanism in breast cancer, *Fusobacterium nucleatum* increased MMP9, but not MMP2, production by breast cancer cells and promoted lung metastasis in preclinical models (47). Collectively, these findings suggest that microbiome-driven activation of EMT, focal-adhesion signaling, and host matrix-degrading enzymes may contribute to ECM remodeling and metastatic progression in breast cancer. Nevertheless, direct evidence that defined breast tumor-resident bacterial species enzymatically degrade collagen, elastin, hyaluronan, or other ECM components *in vivo* is still lacking; microbiome-mediated ECM remodeling should therefore be considered a plausible and emerging mechanism rather than an established one.

Intracellular bacteria have been detected within breast circulating tumor cells (CTCs), suggesting that microbes may “hitchhike” during metastatic dissemination (2, 69). In the MMTV-PyMT spontaneous breast cancer model, Fu et al. showed that breast tumors contain viable low-biomass bacteria, mainly belonging to the genera *Staphylococcus*, *Lactobacillus*, *Enterococcus* and *Streptococcus*. These bacteria were largely detected within the cytosol of tumor cells and their depletion reduced lung metastasis without affecting primary tumor growth. Indeed, these intracellular bacteria can travel along with CTCs and promote metastatic colonization by modulating the Ras homolog family member A (RhoA)-Rho-associated coiled-coil-containing protein kinase (ROCK) signaling pathway and reorganizing the actin cytoskeleton, thereby increasing resistance to mechanical stress in the bloodstream (13), thus supporting distant colonization (12, 13).

An additional mechanism particularly relevant to HR+ breast cancer involves microbial regulation of estrogen metabolism at both systemic and potentially local levels. In the gut, bacterial β-glucuronidases belonging to the estrobolome deconjugate biliary estrogen metabolites, promoting their enterohepatic recirculation and thereby modulating circulating estrogen levels (70). Bacterial taxa with the predicted capacity to produce β-glucuronidase have been identified in breast ductal fluid, raising the possibility that the local microbiota may influence estrogen availability (71). However, bacterial β-glucuronidase activity and estrogen deconjugation have not yet been directly demonstrated within breast tumors.

Systemic dysbiosis may further influence breast cancer progression. For example, gut enterotoxigenic *Bacteroides fragilis* (ETBF) has been shown to promote mammary epithelial hyperplasia, cancer stemness, and metastatic dissemination through activation of β-catenin/Notch1 signaling and induction of a systemic inflammatory response characterized by increased IL-17A and IL-6 production (72). These findings highlight the potential contribution of gut microbiome-derived signals to tumor progression and immune modulation. However, the role of the gut microbiome in breast cancer has been extensively reviewed elsewhere (73–75), and is beyond the scope of the present review, which focuses primarily on the breast microbiome and its interactions with the TIME.

## 4 The interplay between the immune system and the microbiome in breast cancer

As discussed, the breast TME is a complex ecological niche in which malignant cells, stromal components, immune cells, and tumor-resident bacteria co-exist. Although breast tissue is a low-biomass environment, microbial communities are often localized in close proximity to, or even within, both cancer and immune cells (33, 34). Nejman et al. showed that bacteria in breast tumors are mainly intracellular and can be detected not only in cancer cells but also in immune cells, supporting direct spatial proximity between microbes and some immune components of the TME (33). This strict spatial relationship suggests that tumor-resident bacteria may be involved in the regulation of immune responses rather than simply representing passive colonizers. In this context, the breast microbiome can be seen as an additional layer of the TIME, which might influence immune response against the tumor. One of the main ways through which bacteria may affect breast cancer immunity is the activation of PRRs. Bacterial components such as LPS, lipoteichoic acid, peptidoglycan, flagellin and unmethylated CpG DNA can be sensed by innate immune receptors such as TLRs, NOD-like receptors and other sensors expressed by epithelial cells, tumor cells, macrophages, dendritic cells and lymphocytes. In general, activation of PRRs may promote antigen presentation, type I interferon responses and cytotoxic immune activation, or sustain chronic inflammatory pathways such as NF-κB, MAPK and STAT3 (65).

In breast cancer, breast microbiota profiles have been associated with prognostic features and immune activation pathways, suggesting that differences in bacterial composition can contribute to inflammation and immune states observed among patients. Indeed, transcriptional analysis have shown that certain microbial profiles correlate with alterations of immune pathways, including TLR signaling, cytokine profiles and inflammatory gene programs (34), suggesting that intratumoral bacteria may alter immune cell activation and recruitment, affecting tumor development and progression, although the direction and causality of these relationships remain to be established. Consistently, microbial modulation of antitumor immunity has been mechanistically demonstrated in other tumor types: in colorectal cancer, *Fusobacterium nucleatum* can suppress NK- and T-cell-mediated tumor killing through the interaction of its Fap2 protein with the inhibitory receptor TIGIT (76), whereas in pancreatic cancer, tumor-associated bacteria can induce innate and adaptive immune suppression through TLR-dependent mechanisms, and microbial diversity has been associated with immune infiltration and long-term survival (77, 78).

In a large study on fresh-frozen neoplastic and normal breast tissues, Tzeng et al. found that *Propionibacterium* and *Staphylococcus* showed negative associations with oncogenic immune features such as expression of Tumor necrosis factor receptor-associated factor (TRAF)4, whereas *Streptococcus* and *Propionibacterium* positively correlated with genes related to T-cell activation, suggesting that loss of selected commensal taxa may be linked to impaired antitumor immune signaling (34). Using transcriptomic, microbiome, and clinical data from the TCGA-BRCA cohort, Li et al. investigated the relationship between the tumor microbiome and immune heterogeneity in breast cancer. The authors identified two distinct immune subtypes, termed immune-enriched and immune-deficient. The immune-enriched subtype was characterized by higher infiltration of CD8^+^ T cells and M1 macrophages and was associated with a more favorable prognosis. Importantly, significant differences in both α- and β-diversity of the tumor microbiota were observed between the two immune states, indicating a close association between microbial community composition and the TIME. Through differential abundance analysis, 33 immune-related intratumoral microorganisms were identified. Among these, *Acidibacillus, Succinimonas, Lachnoclostridium, and Pseudogulbenkiania* were incorporated into a prognostic microbial signature that effectively stratified patients according to survival outcomes. Furthermore, the abundance of these taxa correlated with immune-related gene expression patterns, the degree of immune-cell infiltration, and predicted responsiveness to chemotherapy agents such as tamoxifen and docetaxel (79). Collectively, these findings suggest that specific components of the breast tumor microbiota are associated with distinct immune phenotypes and might contribute to shaping antitumor immune responses, highlighting their potential value as prognostic biomarkers and therapeutic targets in breast cancer.

Consistently with the association between microbial composition and immune phenotypes, tumor-associated bacteria have also been shown to directly influence immune cell infiltration in breast cancer models. An example is represented by *Fusobacterium nucleatum*. Parhi et al. demonstrated that, once established within the tumor, *F. nucleatum* promoted tumor growth and metastatic dissemination, while reducing the accumulation of tumor-infiltrating T cells. These effects were attenuated by antibiotic treatment, supporting the idea that specific bacteria present in the tumor niche can contribute to a less T cell-infiltrated and more permissive TIME (47). Moreover, previous studies not related to breast cancer showed that *F. nucleatum* can suppress NK- and T-cell cytotoxicity by binding the inhibitory receptor T-cell immunoreceptor with Ig and ITIM domains (TIGIT) via Fap2 protein, suggesting a mechanism by which this bacterium could interfere with antitumor immune responses (76). Additional evidence comes from Xuan et al., who reported reduced expression of anti-bacterial immune response genes in breast tumors compared with healthy breast tissue, together with a lower bacterial DNA load in more advanced disease stages, suggesting that breast cancer progression may be associated with impaired local microbial sensing and immune responsiveness (22).

The complex and context-dependent role of the tumor microbiome in shaping the TIME is suggested by the fact that while some bacterial species appear to promote immunosuppressive and tumor-supportive programs, other strains may enhance antitumor immunity by fostering cytotoxic lymphocyte activity and pro-inflammatory immune cell polarization (**Figure 2**). Indeed, Bernardo et al. recently showed that the mammary tumor microbiome can directly modulate antitumor immunity in a TNBC mouse model. They demonstrated that *Staphylococcus epidermidis* is abundant in breast tumor tissues and can induce a strongly inflamed and tumor-supportive microenvironment, with increased pro-inflammatory cytokine release, complement activation, Treg infiltration and macrophage polarization toward a pro-tumoral M2 phenotype. Oral ampicillin treatment was able to change the tumor microbiota, reducing *S. epidermidis* abundance. This led to a remodeling of the TIME promoting an antitumor immune response and decreasing TLR2 and TLR7 expression and complement C3-fragment deposition, ultimately leading to delayed tumor growth. This effect may be associated with the concomitant enrichment of *Micrococcus luteus*, which induced M1-like macrophage polarization and reduced MDSC infiltration (80). In contrast, Liu et al. recently showed that intratumoral *Staphylococcus* in human and murine tumors is linked to higher CD8^+^ T-cell activity and KLR-related innate-like T-cell signatures in TNBC, a BC subtype in which immune infiltration is clinically relevant and TIL density is associated with prognosis and treatment response. High *Staphylococcus* presence within TNBC has been also associated with macrophage and NK cell polarization toward pro-inflammatory phenotypes. In syngeneic TNBC models, intratumoral *S. aureus* reduced NAD metabolite levels, enhanced granzyme B expression in CD8^+^ TILs, and suppressed tumor growth in a CD8^+^ T-cell-dependent manner. Moreover, *Acinetobacter* and *Dietzia* were inversely associated with FoxP3^+^ TIL density (81). These apparently contrasting effects reported for *Staphylococcus* further suggest that the immunological impact of the breast microbiome may depend on species- or strain-specific properties, microbial community composition, and host-related factors.

**Figure 2:**
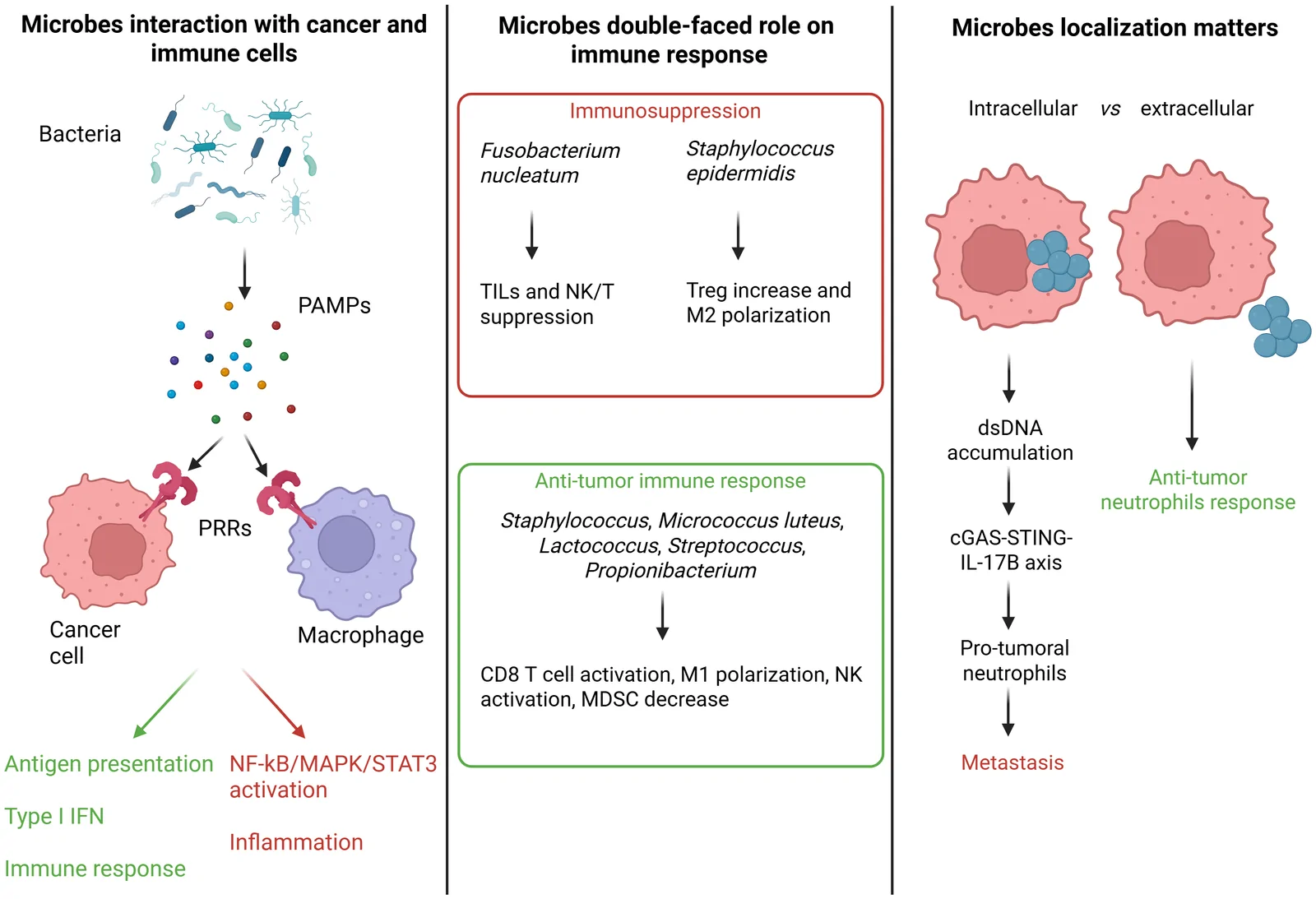
The breast tumor microbiota exerts a context-dependent, dual role in shaping the antitumor immune response. Schematic representation of how the tumor-resident microbiota interacts with the immune system and influences its antitumor activity. Left panel: bacteria located within the tumor release MAMPs that are sensed by pattern recognition receptors (PRRs) expressed on both cancer cells and immune cells, such as macrophages. Depending on the downstream signaling, this recognition can promote antigen presentation, type I interferon (IFN) production and an effective immune response, or drive NF-κB/MAPK/STAT3 activation and inflammation. Central panel: the microbiota plays a double-faced role in the immune response. Immunosuppression is exemplified by *Fusobacterium nucleatum*, which suppresses tumor-infiltrating lymphocytes (TILs) and NK/T-cell activity, and by *Staphylococcus epidermidis*, which promotes regulatory T-cell (Treg) expansion and M2 macrophage polarization. Conversely, an antitumor immune response can be elicited by taxa such as *Staphylococcus, Micrococcus luteus, Lactococcus, Streptococcus* and *Propionibacterium*, which foster CD8^+^ T-cell activation, M1 macrophage polarization, NK-cell activation and a decrease in myeloid-derived suppressor cells (MDSCs). Right panel: the subcellular localization of bacteria also matters. Intracellular bacteria drive double-stranded DNA (dsDNA) accumulation and activation of the cGAS-STING-IL-17B axis, promoting pro-tumoral neutrophils and, ultimately, metastasis, whereas extracellular bacteria elicit an antitumor neutrophil response. Created with Biorender.com.

An additional level of complexity comes from the intracellular localization of bacteria. In the MMTV-PyMT spontaneous breast cancer model, Yao et al. recently showed that the same bacterial composition can shape tumor immunity depending on whether bacteria are localized intra or extracellularly. Intracellular bacteria promoted metastatic recurrence by increasing double-stranded DNA (dsDNA) accumulation, which activates tumor cyclic GMP-AMP synthase (cGAS)-stimulator of interferon genes (STING)-IL-17B axis. The activation of this pathway redirects neutrophils toward a pro-tumoral and immunosuppressive phenotype, which inhibits cytotoxic T-cell activity. Bacterial clearance decreased suppressive neutrophil recruitment and increased CD8^+^ T-cell and NK-cell cytotoxic programs. On the contrary, extracellular bacteria induced a distinct, antitumor neutrophil response, indicating that bacterial localization, and not only bacterial identity or abundance, is important for remodeling of the TIME (82).

Importantly, extracellular bacteria do not invariably exert antitumor effects. Depending on their identity and molecular products, they may persist in the interstitial space, ductal lumina, necrotic or poorly vascularized microniches, or remain associated with the surface of cancer and stromal cells, from which they can promote tumor progression without entering host cells. Thus, extracellular microorganisms can influence the TME through both cell-surface interactions and diffusible microbial products, even when viable bacteria are present at low abundance (54). Importantly, the presence of extracellular microorganisms in the TME should not be interpreted as evidence of systemic infection or a septic condition, which is defined as life-threatening organ dysfunction caused by a dysregulated systemic response to infection (83). Intratumoral microbial communities are generally characterized as low-biomass, spatially restricted ecosystems, with bacterial signals localized to discrete cellular, stromal, vascular, or necrotic niches rather than reflecting uncontrolled systemic bacterial dissemination. Their local abundance may be limited by neutrophils, macrophages, complement, stromal barriers, nutrient availability, and poor vascular perfusion, while hypoxic, necrotic, and immunosuppressive areas may permit their persistence. The antitumor neutrophil response elicited by extracellular bacteria in the MMTV-PyMT model may itself contribute to controlling bacterial expansion (82).

Overall, current evidence suggests that the breast microbiome may influence antitumor immunity. Indeed, tumor-resident bacteria may activate innate immune sensors, regulate inflammatory cytokines, influence antigen presentation and affect the recruitment and activity of NK cells, macrophages, dendritic cells, CD8^+^ T cells, Tregs and suppressive myeloid populations. Depending on the microbial composition, host context and tumor subtype, these interactions may support immune surveillance or promote chronic inflammation, immune suppression and tumor progression. Nevertheless, most human data remain associative, and the field is still limited by the technical challenges of low-biomass microbiota analysis, contamination control, lack of strain-level resolution and limited spatial information. Future studies combining metagenomics, metabolomics, spatial imaging and single-cell immune profiling will be necessary to clarify whether breast-associated microorganisms are causal regulators of antitumor immunity and/or biomarkers of specific immune states.

## 5 The microbiome and therapy resistance in breast cancer

Both gut and tumor-associated microbiota have emerged as an important contributing factor in anti-cancer treatment response across many tumor types, influencing both the efficacy and toxicity of chemotherapy, endocrine therapy and immunotherapy. In breast cancer, this relationship seems to involve both the gut microbiome, which acts systemically through immune, metabolic and inflammatory pathways, and the tumor microbiome, which may directly interact with cancer cells, immune cells and therapeutic agents within the TME (84, 85). The interaction between microbiome and therapy is bidirectional. Indeed, pre-existing microbial communities may influence treatment response, while anti-cancer therapies can reshape microbial composition by reducing beneficial commensals and favoring the expansion of potentially pro-tumoral taxa. This bidirectional interaction may reinforce dysbiosis and may contribute to the emergence of therapy-resistant tumor cell populations (84, 85).

The gut microbiome might influence breast cancer therapy through systemic immune and metabolic mechanisms. In a preclinical TNBC model, depletion of intestinal bacteria with broad-spectrum antibiotics impaired the antitumor activity of doxorubicin, whereas transfer of fecal microbiota from high-fat diet mice promoted tumor growth and reduced doxorubicin efficacy, supporting a role for the intestinal microbiota in regulating systemic and intratumoral immune responses rather than directly degrading the drug (86). Nevertheless, human intestinal bacteria are also capable of directly metabolizing doxorubicin: ex vivo studies demonstrated bacterial deglycosylation and reduction of the drug to 7-deoxydoxorubicin and 7-deoxydoxorubicinol, metabolites with reduced cytotoxic activity (87), and subsequent experiments using simplified intestinal microbial communities confirmed that this biotransformation protects bacterial populations from doxorubicin toxicity (88). However, these studies did not demonstrate that intestinal bacterial metabolism substantially reduces systemic or intratumoral doxorubicin exposure in breast cancer patients. Conversely, gut bacterial metabolism may also enhance the availability of active treatments: bacterial β-glucuronidases hydrolyze glucuronidated endoxifen and 4-hydroxytamoxifen excreted into the intestine, facilitating its enterohepatic recycling, and gnotobiotic and antibiotic-treated mouse experiments showed that the gut microbiota influences circulating concentrations of tamoxifen and its active metabolites (89).

Regarding the breast tumor microbiota, Chiba et al. reported that neoadjuvant chemotherapy (NAC) did not significantly alter the total bacterial load of breast tumors but reduced intratumoral microbial diversity, with a relative enrichment of *Pseudomonas*, while higher *Brevundimonas* and *Staphylococcus* abundance in primary tumors correlated with subsequent distant metastasis. Experimentally, *Pseudomonas aeruginosa* conditioned medium altered breast cancer cell signaling, apoptosis, and chemotherapy responsiveness. These findings suggest that chemotherapy may exert selective pressure on the tumor microbiota and that products released by selected bacterial taxa can modulate breast cancer cell responses to chemotherapy (90). Consistently, pretreatment breast tumor biopsies from patients with TNBC displayed distinct intratumoral microbial profiles according to pathological response: non-pathological complete response (pCR) tumors showed greater microbial diversity and enrichment of Gram-negative taxa, including *Klebsiella*, *Escherichia* and *Acinetobacter*, while LPS reduced the sensitivity of MDA-MB-231 cells to doxorubicin and paclitaxel, potentially through PI3K/AKT reactivation (91). Clinical evidence nevertheless remains predominantly associative: in ER-positive breast cancer, NAC reduced local bacterial density and diversity relative to surgery-only controls, but pretreatment bacterial density did not differ significantly between good and poor responders, indicating that chemotherapy can reshape the breast microbiota without establishing that baseline microbial load determines therapeutic efficacy (92). More direct mechanistic evidence was provided by Ma et al., who detected enrichment of enterotoxigenic *Bacteroides fragilis* (ETBF) in breast tumor tissues from patients resistant to taxane-based NAC. Even at low abundance, tumor-resident ETBF released BFT-1, which bound to and stabilized NOD1 in ALDH-positive breast cancer stem cells, promoted NUMB degradation and activated NOTCH1-HEY1 signaling, thereby increasing stemness and chemoresistance; conversely, bacterial clearance or NOD1 inhibition improved chemotherapy sensitivity in experimental models (93).

Microbiota-associated therapy resistance may also involve immune remodeling. Some chemotherapeutic agents can induce immunogenic cell death, leading to the release of tumor antigens and danger signals that activate dendritic cells and support cytotoxic T-cell responses (94). In this context, altered microbial communities may interfere with therapy-induced antitumor immunity by sustaining inflammatory signals, favoring suppressive immune populations and limiting effective CD8^+^ T-cell activity. This is particularly relevant in TNBC, where the composition and functional state of the TME strongly influence response to neoadjuvant therapy and immune checkpoint blockade. Indeed, the strongest evidence linking the tumor microbiota to therapeutic response has emerged from studies on TNBC. In an exploratory analysis of publicly available TNBC RNA-seq datasets, Kim et al. demonstrated that patients achieving pCR after NAC displayed a significantly different intratumoral microbial composition compared with patients with residual disease. In particular, *Pandoraea pulmonicola* and *Brucella melitensis* were identified as candidate taxa associated with treatment response. Additional candidate taxa, such as *Geosporobacter ferrireducens* and *Streptococcus sanguinis* correlated with increased infiltration of resting NK cells in tumors achieving pCR. Conversely, profiles enriched in *Nitrosospira briensis* and *Plantactinospora* sp. BC1 were associated with higher Treg-cell abundance in residual disease, suggesting that distinct intratumoral microbial communities may be linked to divergent immune landscapes and chemotherapy outcomes (95). However, these findings have not been validated through culture-based, targeted microbial, or spatial validation. More recently, Chen et al. combined 16S rDNA sequencing with single-cell RNA sequencing in patients with early-stage TNBC treated with neoadjuvant chemoimmunotherapy. Tumors achieving pCR harbored a significantly higher intratumoral microbial load and greater bacterial diversity than non-responding tumors. Notably, bacteria were detected within both tumor and immune cells, and increased microbial abundance was associated with enhanced infiltration of CD4^+^CXCL13^+^ T cells and reduced infiltration of immunosuppressive macrophages, suggesting a direct link between the tumor microbiota, treatment response, and local immune activation (96).

The relationship between the tumor microbiota and immunotherapy is particularly intriguing, although still poorly defined in breast cancer. Intratumoral microorganisms may influence both innate and adaptive immune responses, thereby affecting the efficacy of immune checkpoint blockade. For example, *F. nucleatum* colonizes mammary tumors and suppresses antitumor immunity by reducing T-cell activity and impairing cytokine production, while promoting tumor growth and metastatic progression (47). Further evidence indicates that *F. nucleatum* can impair T-cell function and increase PD-L1 expression in a NF-kB-dependent manner, suggesting a possible link between bacterial colonization, immune suppression and sensitivity to immunotherapy (97). Conversely, some microbial metabolites may enhance antitumor immunity. Wang et al. showed that the bacterial metabolite trimethylamine N oxide (TMAO) is enriched in TNBC with an immune-activated TME and can improve the response to immune checkpoint blockade. Mechanistically, TMAO activates the endoplasmic reticulum stress sensor protein kinase R-like endoplasmic reticulum kinase (PERK), triggering gasdermin E (GSDME)-dependent pyroptosis in tumor cells. This immunogenic form of cell death promotes the release of inflammatory mediators that enhance CD8^+^ T-cell activation and effector function, resulting in increased intratumoral infiltration of IFN-γ- and TNF-α-producing CD8^+^ T cells and potentiating the antitumor efficacy of PD-1 blockade in preclinical models. Notably, supplementation with choline, a precursor of TMAO, similarly enhanced the response to immunotherapy, highlighting the therapeutic potential of targeting microbiota-derived metabolites to improve immune checkpoint inhibitor efficacy (38).

Taken together, these findings suggest that microbiota-associated resistance in breast cancer is unlikely to depend on a single mechanism. Rather, it may result from the convergence of treatment-induced microbial selection, bacterial effects on tumor-cell plasticity and stemness, metabolic rewiring and immune remodeling within the TME. In this perspective, the breast cancer microbiota may not be merely a passive component of the TME but might actively influence responses to chemotherapy and immunotherapy. Although most available studies remain exploratory and are largely focused on TNBC, the identification of microbial communities associated with treatment outcomes highlights the potential of the tumor microbiota as both a predictive biomarker and a therapeutic target in precision oncology.

## 6 Therapeutic implications

The identification of a resident microbiota within breast tumors has opened new opportunities for precision oncology. Beyond its plausible contribution to tumor initiation and progression, the tumor microbiome is increasingly recognized as an actionable component of the TME that may be exploited therapeutically or serve as a biomarker. Current strategies can be broadly classified into four approaches: modulation of microbial composition using prebiotics, probiotics or diet, targeting tumor-associated bacteria with antibiotics, exploiting bacteria as therapeutic delivery systems, and using microbial profiles to predict treatment response (**Figure 3**, **Table 2**).

**Figure 3:**
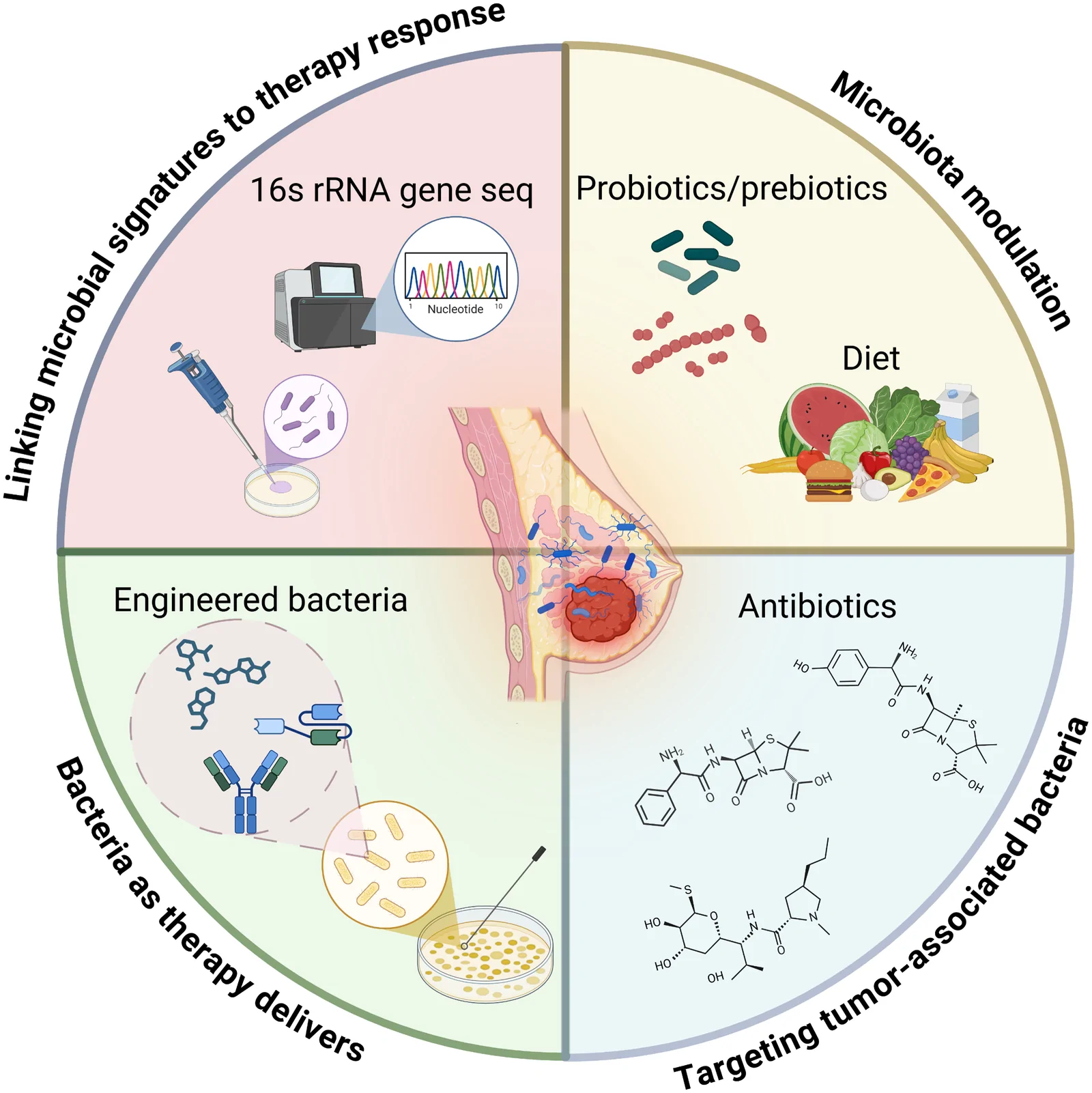
Targeting the tumor microbiota: therapeutic opportunities in breast cancer. Overview of the main approaches through which the breast tumor microbiota can be characterized and therapeutically exploited, each addressing a distinct step of the translational pipeline. Upper left quadrant: culture-based isolation and 16S rRNA gene sequencing allow the identification and taxonomic profiling of the bacteria residing within the tumor, a prerequisite for defining microbial signatures with diagnostic, prognostic and predictive value and for selecting the taxa to be targeted. Upper right quadrant: probiotics, prebiotics and dietary interventions represent non-invasive strategies aimed at restoring a favorable microbial balance, reducing pro-tumorigenic taxa and supporting antitumor immune responses. Lower right quadrant: antibiotics are used to deplete tumor-promoting bacteria; rather than eliminating the whole community, the aim is the selective removal of detrimental species, in order to remodel the TME toward an antitumor phenotype and to improve the response to conventional treatments. Lower left quadrant: bacteria can be engineered to act as living delivery vehicles, exploiting their natural tropism for the tumor to release therapeutic payloads, such as small molecules, cytokines and antibodies, directly within the tumor, thereby achieving high local drug concentrations while limiting systemic toxicity. Created with BioRender.com.

**Table 2:** Therapeutic strategies and translational applications of the breast tumor microbiota (The table is restricted to the therapeutic and translational applications discussed in Section 6; mechanistic aspects addressed elsewhere in the manuscript are not included).

Category	Microorganism/mechanism	Role in breast cancer	Therapeutic strategy	Therapeutic effect	Model evidence stage	Reference
Microbiota modulation (probiotics, prebiotics, diet)	*Lactobacillus acidophilus*	Commensal taxon capable of shifting local immune tone	Oral probiotic supplementation	Slows tumor growth, increases survival; shifts immune response toward Th1 phenotype (↑IFN-γ, ↓IL-4)	Preclinical (rodent)	Imani Fooladi A.A. et al., 2015 (98)
	*Lactobacillus helveticus* (fermented milk)	Restores anti-inflammatory cytokine balance	Dietary/probiotic fermented milk	Decreases IL-6, increases IL-10 in serum and mammary gland	Preclinical (rodent)	de Moreno de LeBlanc A. et al., 2005 (99)
	*Bifidobacterium, Lactobacillus, E. faecalis* (mixed formulation)	Chemotherapy-induced dysbiosis contributes to treatment toxicity	Adjuvant oral probiotic supplementation during chemotherapy	Reduces chemotherapy-associated cognitive impairment	Clinical (randomized controlled trial)	Juan Z. et al., 2022 (101)
	*Staphylococcus aureus*-derived extracellular vesicles	Bacterial EVs modulate hormone-receptor pathway activity	EV-based adjuvant to endocrine therapy	Enhances tamoxifen efficacy in endocrine-sensitive cells	Preclinical (*in vitro*)	An J. et al., 2022 (100)
	Diet-shaped mammary microbiota (Mediterranean vs. Western)	Long-term diet reshapes mammary microbial ecology and local metabolite exposure, independent of the gut (non-tumor-bearing models)	Dietary intervention	~10-fold Lactobacillus enrichment; altered bile-acid/anti-inflammatory metabolites; modulates mammary inflammation and tumor susceptibility	Preclinical (non-human primate, rodent)	Shively C.A. et al., 2018 (102), Arnone A.A. et al., 2024 (103)
Targeting tumor-associated bacteria (antibiotics)	*Staphylococcus epidermidis*	Drives an inflamed, immunosuppressive TME	Oral absorbable antibiotics (ampicillin, clindamycin)	Increases T-cell/M1 infiltration; synergizes with paclitaxel	Preclinical (mouse TNBC model)	Bernardo G. et al., 2023 (80)
	Intracellular bacteria within circulating tumor cells	Promote metastatic colonization	Cell-membrane-penetrating antibiotics (doxycycline, azithromycin)	Reduces lung metastasis without affecting primary tumor growth	Preclinical (mouse model)	Fu A. et al., 2022 (13)
	*Fusobacterium nucleatum*	Immunosuppressive, pro-metastatic tumor colonizer	Biomimetic metronidazole-loaded, tumor-targeted nanovehicle	Selective intratumoral eradication; increases CD8+ infiltration; improved anti-PD-L1 response, with minimal alteration of systemic microbial diversity	Preclinical (mouse model)	Geng S. et al., 2024 (105)
	*Staphylococcus aureus* (intratumoral)	Detectable target guiding antibiotic selection	Bacteria-responsive MRI probe used to screen and identify an active antibiotic (moxifloxacin)	Reduces metastatic nodules versus an ineffective agent	Preclinical (mouse model)	Wu S. et al., 2026 (106)
	Broad-spectrum systemic antibiotic exposure (risk/caution)	Disrupts gut microbiota, impairing systemic antitumor immunity	(Contrast case: non-selective antibiotic use)	Associated with poorer survival and higher recurrence in TNBC patients	Clinical (retrospective cohort)	Ransohoff J.D. et al., 2023 (104)
Bacteria-based delivery systems and bacterial biomimetics	Attenuated *E. coli Nissle 1917*, Salmonella strains (general platforms)	Not validated in breast-tumor models	Engineered to express cytokines, checkpoint inhibitors, or nanobodies	Potentially increases local drug delivery while limiting systemic exposure	Preclinical (proof-of-concept, other tumor types)	Zhou S. et al., 2018 (107), Gurbatri C.R. et al., 2020 (110)
	Engineered *E. coli Nissle 1917* (romidepsin-producing)	Breast-specific application of bacterial drug biosynthesis	EcN engineered to synthesize and deliver the HDAC inhibitor romidepsin	Improves efficacy of bacteria-mediated cancer therapy	Preclinical (murine breast tumor model)	Ma C. et al., 2026 (111)
	Fusobacterium-mimicking nanovehicles	Exploits F. nucleatum’s natural tumor tropism	Biomimetic nanoparticle drug delivery	Enhanced tumor accumulation; improved antitumor efficacy in TNBC	Preclinical (mouse model)	Chen L. et al., 2024 (112)
	Bacterial outer membrane vesicles (OMVs)	Naturally immunostimulatory bacterial nanostructures	Engineered OMVs as dual drug-delivery/immune-activating carriers	Combined payload delivery with innate/adaptive immune stimulation	Preclinical	Liu X. et al., 2023 (113)
	Recombinant *Listeria monocytogenes* (MAGE-B + α-GalCer)	Live bacterial vector platform for antigen delivery	Recombinant bacterial vaccine expressing tumor antigen plus NKT-cell agonist	Enhances cytotoxic immunity; reverses immunosuppression; limits metastasis	Preclinical (mouse model)	Singh M. et al., 2014 (114)
Microbiota-derived diagnostic, prognostic, and predictive biomarkers	Detection toolbox: culture, qPCR/ddPCR, 16S & shotgun sequencing, FISH, spatial sequencing	Measures microbial burden, composition, and cellular/spatial localization, applied by the authors to the breast context	Complementary detection methods with contamination-controlled protocols	Enables robust microbial biomarker discovery in low-biomass tissue	Methodological/translational; enabling methodology, not itself a biomarker	Wang Z. et al., 2025 (115), Xie Y. et al., 2022 (117)
	Multibacterial classifiers (biopsy-derived)	Discrimination between malignant and non-malignant breast tissue	16S rRNA-based diagnostic classifier	Candidate diagnostic biomarker for malignancy status	Translational proof-of-concept study with internal cross-validation (retrospective cohort), diagnostic	Hogan G. et al., 2021 (119)
	*Fusobacterium nucleatum* (tumor colonization)	Colonizes breast tumors; promotes tumor growth, reduces tumor-infiltrating T cells, and increases metastatic dissemination	Candidate prognostic biomarker of tumor growth, immune status, and metastatic risk, supported by preclinical evidence.	Tracks with tumor growth, impaired T-cell activity, and metastatic risk	Preclinical (murine breast cancer model)	Parhi L. et al., 2020 (47)
	*Brevundimonas, Staphylococcus* (primary tumor abundance)	Enrichment in primary tumor correlates with distant metastatic risk	Prognostic biomarker panel	Associated with subsequent relapse/metastasis	Translational (retrospective cohort), prognostic	Chiba A. et al., 2020 (90)
	Subtype-associated microbial diversity (HR+ > HER2+ > TNBC)	Links microbial diversity to molecular subtype and prognosis	Prognostic stratification tool	Refines existing molecular/prognostic classification	Retrospective cohort, prognostic	Banerjee S. et al., 2021 (36)
	Intratumoral microbial abundance + immune profiling (TNBC, NAC-treated)	Reflects treatment-induced microbiota changes	Predictive biomarker for chemo-immunotherapy response	Identifies taxa linked to higher T-cell infiltration/fewer immunosuppressive macrophages; stratifies pCR	Translational cohort, predictive	Chen Y. et al., 2025 (96)

Microbial composition may be modulated using either probiotics or prebiotics. However, while these strategies have been largely developed to manipulate gut microbiota, currently no studies have investigated their effects on breast microbiota. Probiotics are live organisms capable of restoring microbial balance and enhancing antitumor immunity (17). For instance, oral administration of *Lactobacillus acidophilus* has been shown to slow down tumor growth, increase survival rate, and shift the immune response toward a Th1 phenotype, as indicated by increased IFN-γ production and lower IL-4 levels (98). Conversely, *Lactobacillus helveticus*-fermented milk has been shown to decrease pro-inflammatory IL-6 while increasing anti-inflammatory IL-10 in the serum and mammary glands of mice (99).

Beyond their direct antitumor effects, probiotics also play a supportive and adjuvant role in therapy. Extracellular vesicles (EVs) derived from *Staphylococcus aureus* have been shown to enhance the efficacy of tamoxifen (100), and probiotic formulations containing *Bifidobacterium*, *Lactobacillus*, and *Enterococcus faecalis* significantly reduced chemotherapy-associated cognitive deficits (101). Beyond probiotics and prebiotics, dietary interventions represent a non-invasive strategy to modulate the breast microbiota. Although evidence remains limited, preclinical studies suggest that long-term dietary patterns can reshape the microbial ecosystem of the mammary gland independently of the gut. In a non-human primate model, Shively et al. demonstrated that consumption of a Mediterranean diet for 31 months resulted in a mammary microbiota composition distinct from that induced by a Western diet, with an approximately 10-fold enrichment of *Lactobacillus* spp. This microbial shift was accompanied by increased concentrations of bacterial-modified bioactive metabolites, including bile acid derivatives and other microbial products implicated in anti-inflammatory and anti-proliferative signaling, suggesting that dietary habits can influence the local metabolic landscape of the breast through microbiota-dependent mechanisms (102). More recently, experimental studies have further shown that dietary composition shapes the gut microbiota and modulates mammary gland inflammation and tumor susceptibility, supporting the existence of a gut-breast axis through which nutritional interventions may indirectly influence the breast TME (103). However, direct evidence demonstrating that dietary interventions remodel the breast tumor microbiota in patients is still lacking, representing an important area for future investigation.

Antibiotics, by contrast, play a dual and opposing role in breast cancer, offering localized benefits but some systemic risks (12, 20). On the beneficial side, oral absorbable antibiotics, such as ampicillin or clindamycin, can shift the local TIME toward an antitumor phenotype, characterized by higher T cells and M1 macrophage infiltration. Bernardo et al. specifically demonstrated that ampicillin reduces *Staphylococcus epidermidis* in breast tumors, increasing local inflammation and expanding bacterial populations that may promote antitumor immunity, synergizing with chemotherapeutic agents like paclitaxel (80). Similarly, Fu et al. showed that depleting intracellular bacteria within CTCs with cell-membrane-penetrating antibiotics, e.g. doxycycline or azithromycin, significantly reduces lung metastasis of breast cancer without affecting primary tumor growth (13). However, systemic broad-spectrum antibiotic use causes severe gut dysbiosis, impairing antitumor immune response and increasing overall breast cancer risk (20). In TNBC patients, frequent antibiotic prescriptions following diagnosis are associated with significantly poorer survival and higher disease recurrence (104). Therefore, selective microbiota-directed treatment should not aim to render breast tumors completely sterile, because some tumor-associated bacteria may support antitumor immunity, whereas others may promote immune suppression or metastasis. Conventional systemic antibiotics cannot reliably distinguish these populations; therefore, a precision approach would first require identification of viable, clinically relevant bacteria through contamination-controlled sequencing, species-specific qPCR or FISH, and, whenever possible, culture and antimicrobial-susceptibility testing. The antibiotic should then be selected according to bacterial identity, susceptibility, and localization, since the elimination of intracellular bacteria requires cell-penetrating agents, whereas extracellular bacteria may be accessible to non-cell-permeable drugs. Spatial selectivity could be achieved through intratumoral administration or tumor-targeted carriers that concentrate the antibiotic within the breast lesion while minimizing exposure of the gut and other commensal microbiota. In support of this strategy, Geng and colleagues developed a biomimetic metronidazole-containing nanovehicle capable of selectively eradicating intratumoral *F. nucleatum* in a murine breast cancer model without substantially altering systemic microbial diversity, and demonstrated extensive remodeling of the TME, characterized by increased CD8^+^ T-cell infiltration, reduced immunosuppressive signaling, and markedly improved responses to PD-L1 blockade (105). More recently, a bacteria-responsive magnetic resonance imaging probe enabled the detection and quantification of intratumoral *Staphylococcus aureus* in mouse mammary tumors and was used to identify moxifloxacin as an active antibiotic, which reduced metastatic nodules compared with an ineffective agent (106). In the future, narrow-spectrum antibiotics, bacteriophages, bacteriocins, or sequence-specific antimicrobial systems may provide greater species-level selectivity. However, these approaches remain preclinical, and microbiota-directed antibiotics cannot currently be recommended for unselected patients with breast cancer outside controlled studies.

The discovery that bacteria naturally colonize tumors has also stimulated the development of engineered bacterial platforms capable of selectively delivering therapeutic payloads into the TME. Because many bacterial species preferentially accumulate within hypoxic and immunosuppressed tumor regions that are poorly accessible to conventional drugs, they represent attractive vehicles for precision drug delivery (107). Several synthetic biology approaches have been explored using attenuated *Escherichia coli* Nissle (EcN) 1917 or modified *Salmonella* strains engineered to express cytokines, immune checkpoint inhibitors or nanobodies directly within tumors. Although these platforms remain largely preclinical, they offer the possibility of achieving high local drug concentrations while minimizing systemic toxicity (108–110). Their application to breast cancer is beginning to emerge, as recently illustrated by an EcN strain engineered to synthesize and deliver the histone deacetylase inhibitor romidepsin in murine breast tumor models (111). More recently, the breast tumor microbiota itself has inspired biomimetic therapeutic systems. Engineered *Fusobacterium*-mimicking nanovehicles have been developed to exploit the natural tumor tropism of this bacterium for selective delivery of therapeutic agents to TNBC, resulting in enhanced tumor accumulation and improved antitumor efficacy (112). Likewise, bacteria-derived outer membrane vesicles (OMVs) have emerged as highly versatile nanocarriers capable of simultaneously delivering therapeutic molecules and stimulating innate and adaptive immune responses within the TME (113). An alternative strategy exploits live bacteria as therapeutic vaccines. Using recombinant *Listeria monocytogenes* expressing the tumor-associated antigen MAGE-B together with the invariant natural killer T-cell agonist α-galactosylceramide, Singh et al. demonstrated enhanced cytotoxic immune responses, reversal of tumor-induced immunosuppression and significant inhibition of metastatic dissemination in murine breast cancer models (114).

Beyond therapeutic targeting, the breast microbiota is a promising source of diagnostic, prognostic and predictive biomarkers, given the unique microbial landscape found in tumor tissues. However, reliable assessment of the tumor microbiota requires complementary approaches capable of measuring both microbial burden and community composition. Culture-based methods can identify viable microorganisms and enable functional testing, including antimicrobial susceptibility and drug-metabolism assays, but detect only the cultivable fraction of the community. Quantitative PCR and droplet digital PCR targeting bacterial 16S rRNA genes provide sensitive estimates of microbial load, whereas 16S rRNA gene sequencing allows taxonomic profiling at relatively low cost, although its species-level resolution and functional information are limited. Shotgun metagenomic sequencing offers higher taxonomic resolution and can identify microbial genes potentially involved in anticancer-drug metabolism or resistance, while metatranscriptomic approaches may distinguish transcriptionally active microorganisms from residual microbial DNA. Fluorescence *in situ* hybridization, immunohistochemistry and spatially resolved sequencing can further establish the localization of microorganisms within tumor cells, stromal compartments or immune cells (115–117). Because breast tumors are low-microbial-biomass specimens, contamination introduced during tissue collection, processing and sequencing represents a major concern. Appropriate negative controls, matched non-tumor tissues, standardized sampling procedures and, when possible, orthogonal validation are therefore essential (118). Although these techniques are not yet sufficiently standardized for routine clinical applications, integrating microbial load, composition, spatial distribution and functional activity with clinicopathological and treatment-response data could support the identification of microbial biomarkers that could be used in the future for breast cancer diagnosis, prognosis and prediction of therapy response. Indeed, diagnostically, breast tumors display microbial compositions and diversity patterns that can differ from those of healthy breast tissue, frequently with a predominance of Proteobacteria (10), although reported differences in absolute bacterial load are inconsistent and no universally reproducible breast cancer bacterial profile has yet been established. However, beyond individual taxa, multibacterial classifiers derived from breast biopsies have shown the ability to discriminate malignant from non-malignant tissue (119). Prognostically, *Fusobacterium nucleatum* abundance tracks with tumor growth, impaired T-cell activity and metastatic risk (47, 120), while enrichment of *Brevundimonas* and *Staphylococcus* in primary tumors correlates with distant relapse (90). Distinct microbial patterns have also been reported across breast cancer subtypes and linked to clinical outcome, raising the possibility that microbiome information could refine existing prognostic stratification rather than function as a stand-alone marker (36). Predictively, beyond the load- and diversity-based indicators of pCR discussed above, integrating microbial abundance with immune profiling was associated with response to neoadjuvant chemoimmunotherapy and identified microbial features linked to increased T-cell infiltration and reduced immunosuppressive macrophage abundance (96). Notably, NAC itself reshapes the tumor microbiota (90), indicating that microbial composition may dynamically mirror treatment-induced changes rather than represent a static feature.

Collectively, this growing body of evidence suggests that breast tumor microbiota analysis could eventually support diagnosis, prognostic stratification, and prediction of therapeutic response, while guiding microbiota-directed interventions or the selection of antitumor agents less susceptible to microbial inactivation. In parallel, exploiting the microbiota to modulate the TME may offer novel opportunities to enhance conventional therapies, exert direct antitumor effects, and enable targeted drug delivery. However, prospective clinical validation is required before these approaches can be integrated into precision oncology and inform treatment decisions.

## 7 Future perspectives

Although substantial progress has been made in characterizing the breast tumor microbiota, several fundamental questions remain to be solved to translate microbiota studies into clinical precision oncology. One of the main challenges is establishing the origin, dynamics, and functional significance of microorganisms that reside within breast tumors. It remains unclear whether these microbes derive from the gastrointestinal tract, bloodstream, skin, oral cavity, or adjacent breast tissue, and whether they colonize the mammary gland before tumor initiation or are recruited during disease progression. Likewise, little is known about how microbial communities evolve throughout tumor development, metastatic dissemination, or in response to systemic therapies. Indeed, most studies analyzed tumor microbiota at single points, and large longitudinal studies in breast cancer patients are still missing.

A second major challenge is that most studies to date have established only descriptive associations between microbiota profiles or specific bacteria and breast cancer progression, while only a few were able to demonstrate causality. Although several studies have identified microbial patterns associated with breast cancer molecular subtypes, prognosis, immune composition, and treatment response, direct evidence demonstrating that specific microorganisms actively drive breast carcinogenesis, therapeutic resistance, or metastatic progression remains limited. Similarly, the role of the different PRRs and other immune or cancer cell molecules in mediating the interaction between the microbiome and cancer and immune cells needs further investigation, to identify actionable pathways that can be modulated to modify the effect of the tumor microbiome on cancer progression. Future studies should therefore integrate mechanistic analysis using genetically modifiable microbial models, functional perturbation experiments, patient-derived organoids, and *in vivo* models to determine whether tumor-associated microorganisms act as biological drivers or only as opportunistic passengers within the TME.

Methodological standardization also represents a critical prerequisite for clinical translation. Because breast tumors harbor extremely low microbial biomass, microbiome analysis are particularly vulnerable to environmental and reagent contamination, making rigorous negative controls and standardized protocols for tissue collection, processing, sequencing, and bioinformatic analysis essential. Harmonization of clinical metadata and analytical pipelines across institutions will be needed to enable reproducibility and comparison between independent cohorts (121, 122). An additional challenge is represented by the large number of host- and treatment-related variables capable of shaping the tumor microbiota independently of breast cancer biology. Dietary habits, body mass index, age, menopausal status, alcohol consumption, smoking, physical activity, antibiotic exposure, proton pump inhibitors, metformin, probiotics, endocrine therapies, chemotherapy, immunotherapy, and other concomitant medications are all known to influence the composition and function of the human microbiome (123). However, many of these variables are incompletely reported or not systematically incorporated into current breast tumor microbiome studies, limiting the ability to distinguish disease-specific microbial patterns from patient-specific confounding factors. Future prospective studies should therefore include comprehensive clinical, lifestyle, dietary, and pharmacological metadata, enabling patient stratification and multivariable analysis that account for these potential confounders. Standardized reporting of these variables will be essential to improve reproducibility, facilitate comparisons across independent cohorts, and identify robust microbiota-based biomarkers with clinical utility.

The integration of microbial profiling with complementary molecular and spatial technologies may advance our understanding of the tumor microbiota, favoring its translation into clinical practice. Combining metagenomics, metatranscriptomics, metabolomics, spatial transcriptomics, proteomics, and single-cell analysis will enable simultaneous characterization of microbial communities, host cellular states, and metabolic interactions. The application of artificial intelligence and machine learning combined with these approaches will provide important insights into how microorganisms influence immune modulation, metabolic reprogramming, angiogenesis, epithelial plasticity, and therapeutic response, and will reveal the spatial organization of bacteria within distinct tumor niches.

Thus, the tumor microbiota has the potential to become an additional layer of information for precision oncology. Microbial profiles may complement existing genomic, transcriptomic, and immunological biomarkers to improve patient stratification, predict response to chemotherapy, endocrine therapy, targeted agents, and immunotherapy, and identify patients at increased risk of recurrence or metastatic dissemination. At the same time, therapeutic manipulation of the tumor microbiota, including selective bacterial depletion, the use of engineered bacteria for targeted drug delivery or tumor lysis, the application of bacteriophages to eliminate specific pro-tumorigenic strains, the exploitation of bacterial extracellular vesicles as non-invasive biomarkers or therapeutic agents and the modulation of microbiota-derived metabolites, offers promising opportunities to remodel the TME and enhance the efficacy of existing anti-cancer treatments. Ultimately, large, prospective, multicenter longitudinal studies integrating standardized biospecimen collection, comprehensive clinical and lifestyle metadata, spatial microbiology, multi-omics analysis, and mechanistic validation will be essential to establish causal relationships, identify clinically robust microbial biomarkers, and translate microbiota-based strategies into routine breast cancer management.

## 8 Conclusions

The discovery of a resident microbiota within breast tumors has expanded our understanding of breast TME. Accumulating evidence suggests that tumor-associated bacteria may influence multiple hallmarks of breast cancer, including immune regulation, metabolic reprogramming, epithelial cell proliferation and plasticity, metastatic dissemination, and response to systemic therapies. At the same time, advances in sequencing technologies and spatial profiling have demonstrated that microbial communities are associated with clinically relevant tumor features, supporting their potential as diagnostic, prognostic, and predictive biomarkers.

Despite these advances, the study of the breast tumor microbiota is still in its early stages. In order to translate current discoveries into clinical applications, further studies aimed at investigating the role played by the microbiome in cancer onset and progression and deciphering the functional interactions between microorganisms, tumor and immune cells, are needed. Moreover, efforts to overcome the technical challenges inherent to low-biomass microbiome analysis will be essential. If these challenges can be addressed, integrating microbial profiling with genomic, transcriptomic and immunological analysis may help to further advance precision oncology and develop new strategies for patient diagnosis and stratification. In parallel, the development of innovative microbiota-targeted therapies may provide new strategies for more effective and personalized cancer treatment.
